# Fasting requires Peroxiredoxin 2 and Peroxiredoxin 6 to coordinate redox dependent mitochondrial and lipid remodelling in *Caenorhabditis elegans*

**DOI:** 10.1016/j.redox.2026.104231

**Published:** 2026-05-22

**Authors:** Penglin Li, Yating Zheng, Jose C. Casas-Martinez, Qin Xia, Antonio Miranda-Vizuete, Katarzyna Goljanek-Whysall, Brian McDonagh

**Affiliations:** a Discipline of Physiology, School of Pharmacy and Medical Sciences, University of Galway, Ireland; b Galway RNA Research Cluster, Ireland; c Institute for Health Discovery and Innovation, University of Galway, Ireland; d Department of Orthopaedics, Tongji Hospital, Tongji Medical College, Huazhong University of Science and Technology, China; e Instituto de Biomedicina de Sevilla, IBiS/Hospital Universitario Virgen del Rocío/CSIC/Universidad de Sevilla, Spain; f Institute of Life Course and Medical Sciences, University of Liverpool, UK

**Keywords:** Fasting, Peroxiredoxin, Mitochondrial dynamics, Lipid remodelling, Oleic acid, Ageing

## Abstract

Fasting induces conserved metabolic and redox adaptations that promote stress resistance and longevity. However, the molecular mechanisms linking transient redox changes and altered metabolism to downstream signalling events remain incompletely understood. Using *Caenorhabditis elegans*, the roles of peroxiredoxins in coordinating redox-dependent responses to fasting and refeeding were determined. A 4-hr fasting protocol over 5 days extended lifespan, improved late-life physiological activity, reduced age-related lipofuscin and lipid accumulation. The fasting protocol generated a transient increase in mitochondrial ROS, promoted mitochondrial turnover, and attenuated age-related mitochondrial fragmentation. These adaptive responses required the activation and nuclear localisation of the stress-responsive transcription factors DAF-16/FOXO and SKN-1/Nrf2. However, these adaptive responses were abolished in *prdx-2* and *prdx-6* mutant strains, which exhibited persistent redox imbalance, mitochondrial fragmentation, altered stress resistance, and disrupted DAF-16 and SKN-1 signalling. Mechanistically, loss of 2-Cys PRDX-2 impaired activation of the p38 MAPK PMK-1 pathway, resulting in defective SKN-1 activation. In contrast, loss of 1-Cys PRDX-6 disrupted lipid metabolic signalling, preventing induction of NHR-80 and downstream fatty acid desaturases required for metabolic adaptations. Despite distinct initial signalling pathways, both peroxiredoxins converged on the regulation of DAF-16 and SKN-1. Together, these findings identify PRDX-2 and PRDX-6 as redox sensors that translate a fasting-induced transient ROS signature into mitochondrial and lipid remodelling pathways to promote healthy ageing.

## Highlights

• 5-day intermittent fasting promoted increased longevity, physiological fitness and filamentous mitochondria in *C. elegans.*
• Fasting triggered transient mitochondrial ROS resulting in mitochondrial network and lipid remodelling.
• 2-Cys PRDX-2 was required for p38 PMK-1 mediated SKN-1 activation following intermittent fasting.
• 1-Cys PRDX-6 was required for NHR-80-dependent lipid remodelling following intermittent fasting.
• Peroxiredoxins translate transient ROS generation to coordinate adaptive signalling responses.

## Graphical abstract

**Figure:**
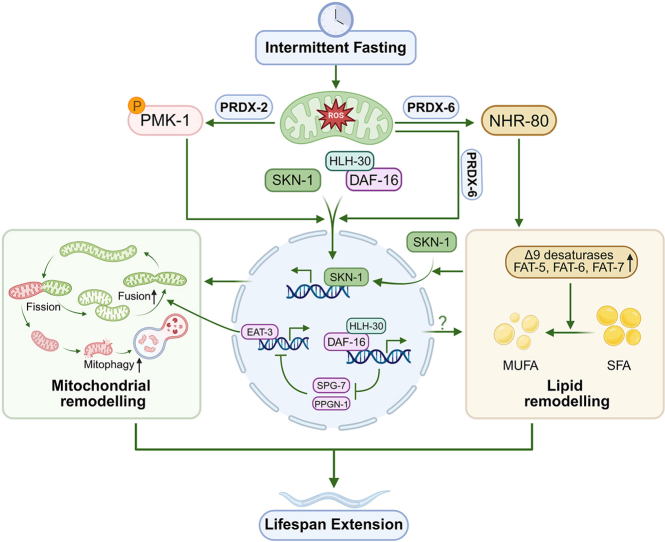

## 1 Introduction

Fasting and dietary restriction have been demonstrated to have a positive impact on longevity and stress resistance in *Caenorhabditis elegans* and several other model organisms [1]. Nutrient availability is a key regulatory factor during development and the deprivation of food results in metabolic remodelling, changes in gene transcription, protein turnover and organelle dynamics [[2], [3], [4]]. *C. elegans* is a powerful model to identify the molecular mechanisms underlying the metabolic adaptations to fasting based interventions in higher organisms. Induction of metabolic stress as a result of nutrient deprivation results in alterations in mitochondrial metabolism, requiring a switch to alternative fuel sources but also involves subsequent metabolic adaptations following refeeding [2,5]. It has been demonstrated that during fasting, mitochondria have a more fragmented morphology related to decreased reliance on glucose as a metabolic substrate, with increased β-oxidation and lipid remodelling [2,6]. Following refeeding in *C. elegans*, the mitochondrial network becomes more elongated, forming a more tubular network [2]. The mechanisms underlying the changes in mitochondrial remodelling during fasting involve reduced insulin/IGF-1 like signalling (IIS) and the nuclear translocation of the transcription factors DAF-16/FOXO and SKN-1/Nrf2 [[7], [8], [9], [10], [11], [12]]. Mammalian FOXO1, FOXO3 and FOXO4 are the orthologs of DAF-16, while SKN-1 is the ortholog of mammalian Nrf2; together these transcription factors regulate the adaptive response to cellular stress. Moreover, both SKN-1 and DAF-16 regulate genes that coordinate mitochondrial dynamics and metabolism [8,13,14].

Fasting and nutritional stress have also been demonstrated to promote changes in the intracellular redox environment, which typically return to basal levels following refeeding [[15], [16], [17]]. Moreover, treatment of *C. elegans* with N-acetylcysteine (NAC) can block the increased generation of reactive oxygen species (ROS) and the associated lifespan extension under conditions of glucose restriction, highlighting a key role of redox signalling in this adaptive response [15]. The adaptations associated with acute ROS generation during fasting are similar to what has previously been reported in *C. elegans* during exercise, with increased DAF-16 nuclear localisation and mitochondrial remodelling [7,8]. Increased DAF-16 nuclear localisation following elevated intracellular ROS levels has also been demonstrated in long lived mitochondrial mutant strains [18]. Energetic stress as a result of fasting or exercise requires metabolic rewiring and determines the balance between mitochondrial fission and fusion [2]. Normally under nutrient replete conditions DAF-16 is phosphorylated by AKT and SGK kinases resulting in its cytoplasmic sequestration by 14-3-3 proteins [9]. Interestingly DAF-16 has increased nuclear localisation in response to acute physiological stress with increased endogenous ROS generation, such as exercise, regulating genes involved in mitochondrial remodelling [7,8]. DAF-16 operates in close cooperation with the TFEB ortholog HLH-30, co-regulating the expression of many target genes, required to promote the organism's stress response [19]. Phosphorylated HLH-30 is also sequestered in the cytoplasm by 14-3-3 proteins and its nuclear localisation under stress conditions has been suggested to be redox dependent [20]. SKN-1 regulates a range of oxidative stress responses in *C. elegans* [21]. In response to elevated stress and increased intracellular ROS, SKN-1 is usually phosphorylated and activated by the p38 PMK-1 pathway [22]. Similar to DAF-16, under conditions of reduced IIS, there is elevated SKN-1 nuclear localisation, which is required for the increased longevity following nutrient deprivation [[10], [11], [12]]. However, it remains uncertain how an initial increase in endogenous ROS generation during altered metabolism is sensed and transmitted to target proteins.

Peroxiredoxins are a family of highly abundant thiol based antioxidant proteins and widely conserved enzymes [23]. Originally identified due to their peroxidase activity, they have also been identified as key regulators of cell signalling cascades, particularly in conditions with a disrupted redox environment or following endogenous ROS generation. Members of the peroxiredoxin family and their redox state have been implicated in regulating yeast metabolic cycling [24], involved in transferring oxidative equivalents to target proteins in a redox relay mechanism [25] and as molecular chaperones [26]. Mammalian species have 6 peroxiredoxin isoforms that have specific cellular locations, while the nematode *C. elegans* has 3 peroxiredoxins, 2-Cys PRDX-2 and PRDX-3 and the 1-Cys Peroxiredoxin (PRDX-6). It has been previously demonstrated that PRDX-2 is required for lifespan extension following metformin treatment and the adaptive response to physiological stress such as during exercise [27,28]. The highly conserved mammalian 1-Cys PRDX6, has a number of different catalytic activities including reducing phospholipid hydroperoxides, Ca^2+^ independent phospholipase and lysophosphatidylcholine acyltransferase activities [29] and its role in lipid remodelling and protection against ferroptosis has been described [30]. Furthermore, the hyperoxidation of peroxiredoxins in *C. elegans* has also been linked to a decline in the redox stress response capacity with age and disrupted DAF-16 activation [7,31].

Many of the adaptive responses to fasting-refeeding cycles and conditions of acute stress are redox dependent and result in both DAF-16 and SKN-1 nuclear localisation, with subsequent mitochondrial remodelling. Previously it was demonstrated that PRDX-2 is a key regulator of the intracellular redox environment and required for the mitochondrial remodelling following acute stress, with disrupted DAF-16 nuclear localisation and altered mitochondrial morphology in *prdx-2* mutants [7]. In this study, the roles of PRDX-2, PRDX-3 and the 1-Cys PRDX-6 in response to fasting were investigated. The results demonstrate that fasting promoted longevity, stress resistance and improved physiological activity, these adaptive responses required DAF-16 nuclear localisation and SKN-1 activation. Following fasting there was extensive mitochondrial remodelling, that was absent in the *prdx-2* and *prdx-6* mutants. Mechanistically, the data presented highlight that the altered fasting response in the *prdx-2* mutant was due to an inability to activate p38 PMK-1 signalling. However, the *prdx-6* mutant had disrupted lipid signalling following fasting and did not increase the expression levels of *nhr-80* and the downstream fatty acid desaturases. The data highlight that under conditions of fasting that resulted in an altered cellular redox environment, the adaptive response was dependent on the highly conserved and abundant peroxiredoxin proteins, PRDX-2 and PRDX-6, although via distinct metabolic pathways that ultimately converged on DAF-16 and SKN-1 activation.

## 2 Results

### 2.1 5-day intermittent fasting (IF) promotes longevity and physical activity with age

**Fig. 1:**
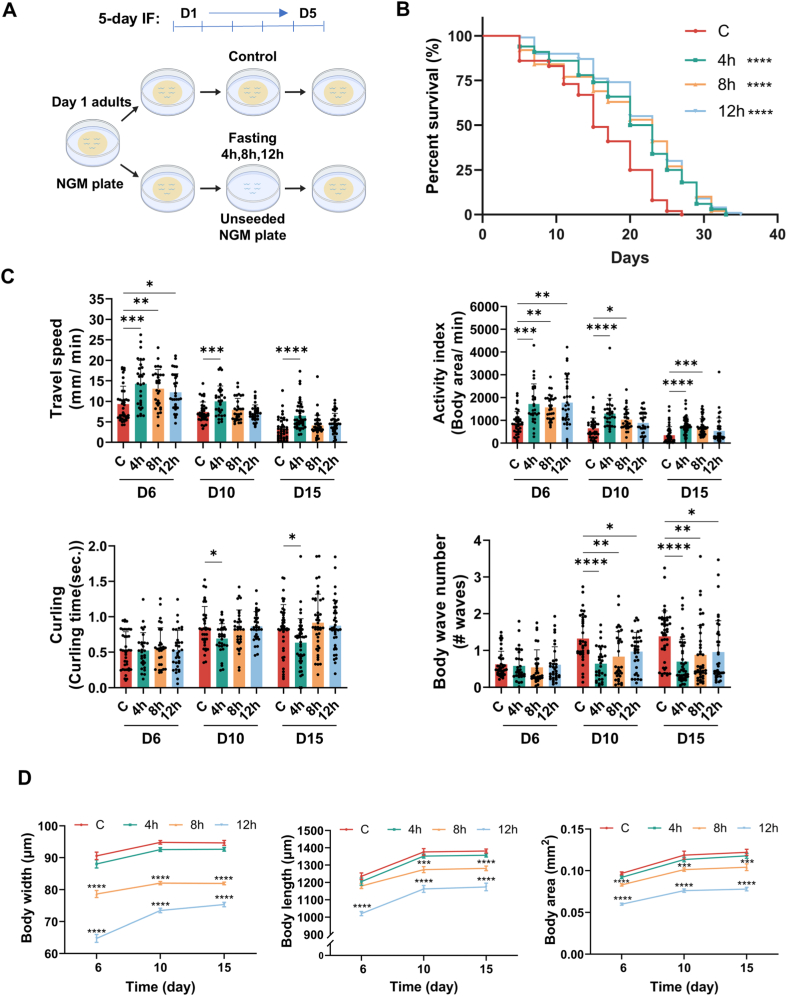
5-day intermittent fasting promotes longevity and preserves physical fitness during ageing in *C. elegans*. (A) Schematic diagram of the 5-day intermittent fasting (IF) protocol initiated in synchronised day 1 adult worms and continued for 5 days. **(B)** Lifespan of wild type N2 strain following the 5-day IF protocol. Kaplan–Meier survival plots of three independent experiments initiated with 105 animals per group. ∗∗∗∗p ≤ 0.0001 compared to the Control (C). **(C)** Assessment of physiological activity (Travel speed, Activity index) and frailty (Curling time, Body wave number) using the CeleST platform on days 6, 10, and 15 following the 5-day IF. ∗p ≤ 0.05, ∗∗p ≤ 0.01, ∗∗∗p ≤ 0.001, and ∗∗∗∗p ≤ 0.0001 (n = 30-45). **(D)** Measurement of morphological parameters including body width, body length, and body area at days 6, 10, and 15 following the 5-day IF. ∗∗∗p ≤ 0.001 and ∗∗∗∗p ≤ 0.0001 (n = 30).

The effects of fasting and dietary restriction on longevity in *C. elegans* have previously been demonstrated [4,32]. In this study, a 5-day fasting protocol was performed with fasting for 4 h, 8 h or 12 h on each day (Fig. 1A). As expected, the fasting protocol increased longevity with no observable differences in terms of the fasting duration (Fig. 1B). Following the initial 5-day fasting protocol, physiological activity was determined on days 6, 10 and 15 using CeleST software [33] (Fig. 1C). There was an age-dependent decrease in travel speed, however all three fasting durations promoted activity on day 6, but only the 4 h fasting duration resulted in increased travel speed on days 10 and 15. Similarly, there was an age dependent increase in frailty parameters such as curling, and the 4 h fasting duration had the most beneficial effect. These results were compared with a single fasting intervention on adult day 1, which also increased lifespan with the 12 h fasting having the most pronounced effect (Fig. S1A). However, single fasting did not improve physiological activity as determined by CeleST on days 6, 10 or 15 (Fig. S1B). There was a significant decrease in body width, length and area following the 5-day 8 h and 12 h fasting protocols in worms on day 6, day 10 and day 15, that was not observed following the 4 h fasting protocol (Fig. 1D, Fig. S1C). The data demonstrates that a 4 h 5-day fasting intervention was the most effective for increased longevity and maintaining physiological activity at days 10 and 15, while not causing a significant decrease in body size.

### 2.2 5-day intermittent fasting promotes mitochondrial remodelling that is maintained with age

**Fig. 2:**
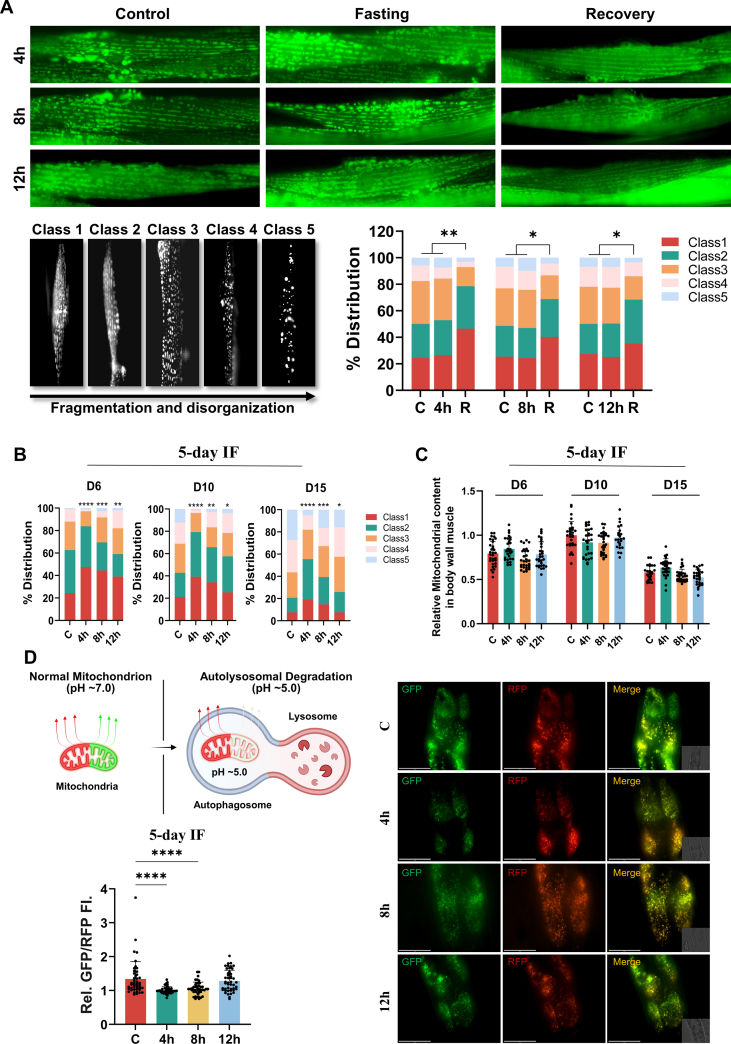
5-day intermittent fasting promotes mitochondrial remodelling and maintains mitophagy during ageing. (A) Representative images of day 1 adults *zcIs14[myo-3p::GFP(mit)]* mitochondrial reporter strain across a pre-fasting (Control, C), fasting (4 h, 8 h, or 12 h), and recovery (R, 16 h) cycle. Black and white representative images of the classification of mitochondrial morphology in body wall muscle. ∗p ≤ 0.05 and ∗∗p ≤ 0.01 (130-150 images per group). **(B)** Quantification of mitochondrial morphological classes of the *zcIs14[myo-3p::GFP(mit)]* mitochondrial reporter on days 6, 10, and 15 following the 5-day IF. ∗p ≤ 0.05, ∗∗p ≤ 0.01, ∗∗∗p ≤ 0.001, and ∗∗∗∗p ≤ 0.0001 compared to control (130-150 images per group). **(C)** Relative mitochondrial content in body wall muscle on days 6, 10, and 15 following the 5-day IF (n = 30). **(D)** Evaluation of mitophagy in day 6 adults following the 5-day IF using the *unc-119(ed3)*; *Ex[myo-3p::tomm-20::Rosella; unc-119(+)]* dual-fluorescence reporter strain. Representative images of muscle specific area of head region just anterior to the gut. Schematic representation of the Rosella biosensor, where GFP is quenched but RFP is stable in the acidic lysosomal environment. Representative fluorescence images of GFP, RFP, and merged channels (Scale bars: 50 μm). ∗∗∗∗p ≤ 0.0001 (n = 45).

Fasting has previously been reported to induce substantial mitochondrial remodelling in *C. elegans* [2]. Decreased nutrient availability results in a switch in the metabolic fuel substrate use, promoting a shift towards β oxidation [5]. To capture the mitochondrial dynamics in response to intermittent fasting, mitochondrial morphology within body wall muscle was monitored using the mitochondrial GFP reporter *zcIs14[myo-3p::GFP(mit)]* across a fasting and refeeding cycle. Immediately following fasting there were only slight changes in muscle mitochondrial morphology but increased filamentous mitochondria after a recovery period, highlighting the extensive metabolic changes during decreased nutrient availability (Fig. 2A). The long-term effects of the early 5-day fasting protocol on mitochondrial morphology during ageing was determined. In the non-fasted controls, there was an age-dependent increase in mitochondrial fragmentation in body wall muscle (Fig. 2B, Fig. S2A). The 5-day fasting protocol resulted in increased filamentous mitochondria on day 6 for all fasting durations, however the 4 h fasting intervention helped prevent age-related mitochondrial fragmentation and this group maintained increased filamentous mitochondria on days 10 and 15 (Fig. 2B, Fig. S2A). Interestingly a single fasting intervention on day 1 for 4 h improved mitochondrial morphology on day 5 but this was not maintained with age and the morphology was similar to controls on days 10 and 15 (Fig. S2B). There was an age-related decrease in muscle mitochondrial content on day 15 but no change between any of the fasting conditions (Fig. 2C, Fig. S2C). One of the key determinants of mitochondrial biogenesis and dynamics is mitophagy. The effect of fasting on mitophagy was assessed in muscle using the *unc-119(ed3); Ex[myo-3p::tomm-20::Rosella; unc-119(+)]* strain. The Rosella biosensor consists of a pH-stable RFP fused with a pH-sensitive GFP. During mitophagy, as mitochondria undergo transportation to the acidic lysosomal environment, the GFP fluorescence undergoes quenching, while the RFP fluorescence remains stable (Fig. 2D) [13]. There was increased mitophagy following the fasting interventions for 4 h and 8 h for 5 days (Fig. 2D), however a single fasting for 8 h and 12 h resulted in decreased mitophagy (Fig. S2D). Together the results highlight the plasticity of mitochondria in response to nutrient availability and that a 4 h fasting over 5 days induces substantial mitochondrial remodelling and prevents age-related mitochondrial fragmentation.

### 2.3 Fasting results in increased DAF-16 nuclear localisation and SKN-1 activation

**Fig. 3:**
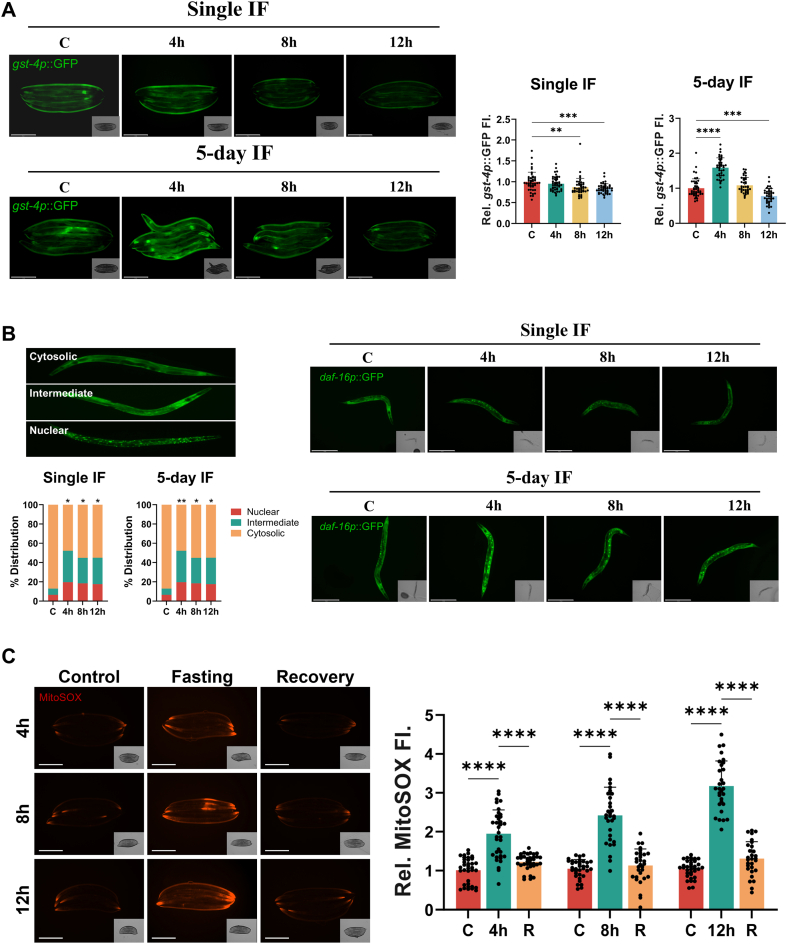
Intermittent fasting induces SKN-1 activation and DAF-16 nuclear localisation mediated by transient mitochondrial ROS generation. (A) Representative images of SKN-1 activation reporter *dvIs19[(pAF15)gst-4p::GFP::NLS] III* acquired from day 2 adults following single IF and from day 6 adults following 5-day IF. (Scale bars: 275 μm). ∗∗p ≤ 0.01, ∗∗∗p ≤ 0.001, and ∗∗∗∗p ≤ 0.0001 (n = 30-45). **(B)** Representative images of DAF-16 nuclear localisation using the *zIs356[daf-16p::daf-16a/b::GFP + rol-6(su1006)]* reporter strain following single and 5-day IF protocols. Images were acquired from day 2 adults after single IF and from day 6 adults after 5-day IF. (Scale bars: 275 μm). ∗p ≤ 0.05 and ∗∗p ≤ 0.01 (n = 30). **(C)** Representative images of mitochondrial ROS across a fasting and refeeding (16 h) cycle performed on day 1 adults in wild type strain (Scale bars: 275 μm). ∗∗∗∗p ≤ 0.0001 (n = 30).

In order to determine the mechanisms underlying the changes in mitochondrial remodelling and dynamics, reporter strains for key molecular signalling pathways such as the UPR, DAF-16 and SKN-1 were used. Reporter strains for UPR^ER^ and UPR^mt^ demonstrated no change following either single or a 5-day fasting protocol at any of the time points (Fig. S3A and B). To assess SKN-1 activation, the SKN-1 reporter strain *dvIs19[(pAF15)gst-4p::GFP::NLS] III* was used. A single fasting event for either 8 h or 12 h resulted in decreased SKN-1 activation, while a 5-day fasting for 4 h duration resulted in robust SKN-1 activation (Fig. 3A). The 4 h 5-day fasting protocol robustly increased SKN-1 activation is consistent with its effects on mitophagy (Fig. 2D). In contrast using *zIs356[daf-16p::daf-16a/b::GFP + rol-6(su1006)]* reporter strain for DAF-16 nuclear localisation, both a single and 5 days fasting intervention resulted in increased DAF-16 nuclear localisation at all time points (Fig. 3B). These results demonstrate that fasting results in robust DAF-16 nuclear localisation, but SKN-1 activation was dependent on the frequency and duration of the nutritional stress. As both SKN-1 and DAF-16 activation have been reported to be dependent on a redox signalling cascade [18,22,34], changes in mitochondrial ROS generation were assessed using MitoSOX staining. Fasting resulted in increased MitoSOX staining that returned to baseline levels following a 16 h recovery period (Fig. 3C). However, incubation of *C. elegans* with 5 mM NAC not only prevented the fasting induced ROS generation but also the increase in filamentous mitochondria following a recovery period (Fig. S3C and D). Moreover, incubating worms in plates containing 5 mM NAC prevented the increased DAF-16 nuclear localisation and SKN-1 activation induced by the 4 h 5-day fasting protocol (Fig. S3E and F). Together the results highlight an acute endogenous ROS generation following fasting, results in SKN-1 activation and DAF-16 nuclear localisation.

### 2.4 Fasting results in distinct changes in genes regulating mitochondrial morphology and redox environment

**Fig. 4:**
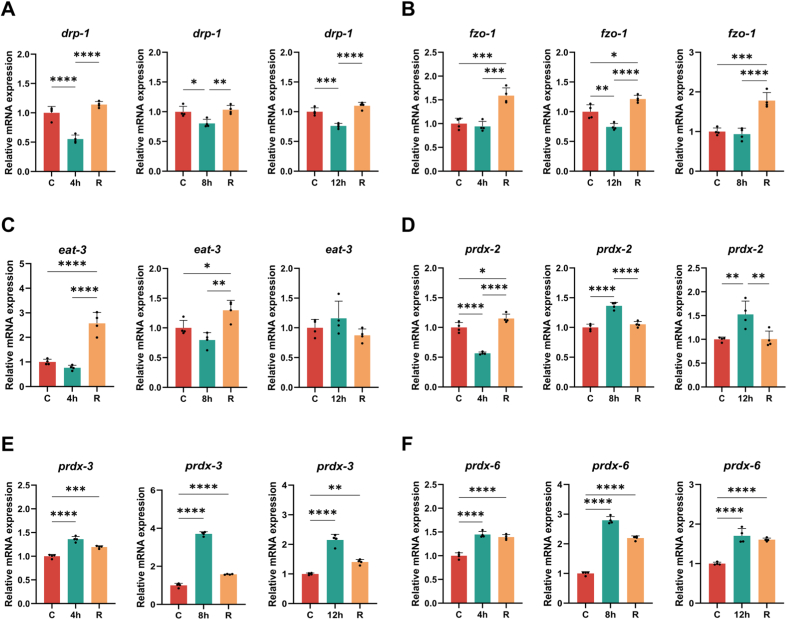
Fasting induces distinct transcriptional changes in genes regulating mitochondrial morphology and the redox environment. (A-C) Relative mRNA expression levels of the mitochondrial fission gene *drp-1*, and fusion genes *fzo-1* and *eat-3* in N2 wild type strain under either control, fasting, and recovery (16 h) cycle starting at adult day 1 (n = 4). **(D**–**F)** Relative mRNA expression levels of the peroxiredoxin genes *prdx-2*, *prdx-3*, and *prdx-6* in N2 wild type under control, fasting, and recovery (16 h) cycle starting at adult day 1 (n = 4). ∗p ≤ 0.05, ∗∗p ≤ 0.01, ∗∗∗p ≤ 0.001, and ∗∗∗∗p ≤ 0.0001.

As the fasting-refeeding cycle results in rapid changes in mitochondrial morphology, the expression levels of genes regulating mitochondrial fission (*drp-1*) and fusion (*eat-3* and *fzo-1*) were analysed by qPCR. The expression levels of *drp-1* significantly decreased immediately following 4, 8 and 12 h of fasting but returned to baseline levels following recovery (Fig. 4A). The expression levels of *fzo-1* increased following a recovery period after 4, 8 and 12 h of fasting, however expression levels decreased immediately following 12 h of fasting only (Fig. 4B). The expression levels of *eat-3* did not significantly change immediately following fasting but increased following recovery after the 4 h fasting (Fig. 4C). The decrease in the mitochondrial fission related gene *drp-1* during fasting, and increased expression levels of fusion related genes (*eat-3* and *fzo-1*) following recovery aligns with the mitochondrial morphological changes detected (Fig. 2A). Furthermore, NAC blocked the fasting induced increase in expression levels of *eat-3* and *fzo-1* following recovery but had no effect on *drp-1* levels (Fig. S3G). Mutant strains of *drp-1*, *fzo-1* and *eat-3* were subsequently subjected to the 4 h 5-day fasting protocol, with longevity and stress resistance analysed. In these mutant strains, fasting did not result in any beneficial adaptations as compared to the wild type strain and there was decreased stress resistance following fasting in the *fzo-1* mutant strain (Fig. S4).

There was an acute increase in mitochondrial ROS during fasting (Fig. 3C) and peroxiredoxins are considered the primary scavengers of H_2_O_2_ [35]. *C. elegans* possess 3 peroxiredoxin isoforms, PRDX-2, PRDX-3 and the 1-Cys PRDX-6. It has been previously demonstrated that PRDX-2 is required for the adaptive response to exercise following endogenous ROS generation [28]. Moreover, PRDX-2 has been linked to SKN-1 activation and insulin dependent signalling in *C. elegans* [36,37]. Considering the transient changes in MitoSOX staining during and following fasting (Fig. 3C), the expression levels of *prdx-2*, *prdx-3* and *prdx-6* were determined. Following 4 h of fasting the expression levels of *prdx-2* decreased but increased following 8 and 12 h of fasting, with levels returning to baseline following recovery (Fig. 4D). At the protein level, there were no changes in overall PRDX-2 abundance but the redox state of PRDX-2 (% as Dimer:Monomer) increased following fasting as detected by non-reducing gel electrophoresis (Fig. S5). In contrast, both *prdx-3* and *prdx-6* showed increased expression levels following all the fasting durations, but *prdx-6* levels in particular maintained elevated expression following recovery (Fig. 4E and F). The data revealed considerable differences in the changes in peroxiredoxin gene expression levels that were dependent both on the duration of the nutritional stress and recovery, with *prdx-2* having very distinct changes compared to *prdx-3* and *prdx-6* especially following 4 h of fasting.

### 2.5 Fasting in *prdx-2* and *prdx-6* mutants results in distinct changes in longevity and stress resistance

**Fig. 5:**
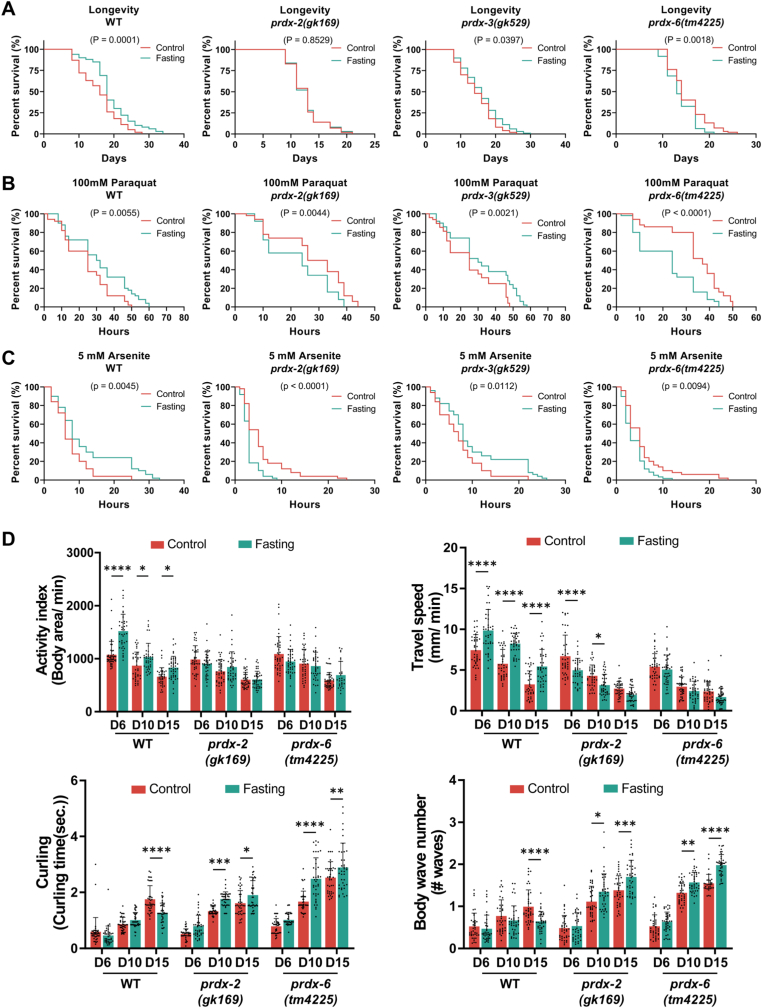
PRDX-2 and PRDX-6 are required for fasting-induced longevity, stress resistance, and the maintenance of physical activity during ageing. (**A**) Lifespan of wild-type N2, *prdx-2(gk169)*, *prdx-3(gk529)*, and *prdx-6(tm4225)* mutant strains subjected to a 4 h 5-day IF protocol compared to controls. Kaplan–Meier survival plots represent experiments initiated with 105 animals per group. P-values are indicated on the graphs. Kaplan–Meier survival plots of day 6 adult wild type N2, *prdx-2(gk169)*, *prdx-3(gk529)*, and *prdx-6(tm4225)* mutant strains in response to paraquat (**B**) and sodium arsenite (**C**) following the 4 h 5-day IF protocol (n = 50). P-values are indicated on the graphs. **(D)** Physiological activity (Travel speed, Activity index) and frailty (Curling time, Body wave number) monitored by CeleST at days 6, 10, and 15 for wild type N2, *prdx-2(gk169)*, and *prdx-6(tm4225)* mutants following the 4 h 5-day IF. ∗p ≤ 0.05, ∗∗p ≤ 0.01, ∗∗∗p ≤ 0.001, and ∗∗∗∗p ≤ 0.0001 (n = 35-45).

As the expression levels of *prdx-2*, *prdx-3* and *prdx-6* varied following nutritional stress and it has previously been described that PRDX-2 was required for adaptive redox signalling including appropriate DAF-16 nuclear localisation [7], *prdx-2*, *prdx-3* and *prdx-6* mutant strains were subjected to the 4 h 5-day fasting protocol. In the wild type and *prdx-3* mutant, fasting resulted in extended lifespan (Fig. 5A). However, fasting in the *prdx-2* mutant had no effect on longevity and resulted in decreased longevity in the *prdx-6* mutant (Fig. 5A). Using the mitochondrial redox cycler paraquat and inducer of cytoplasmic oxidative stress sodium arsenite, stress resistance was determined. Fasting also resulted in increased stress resistance in both the wild type and *prdx-3* mutant strains, however the *prdx-2* and *prdx-6* mutant strains had decreased stress resistance compared to non-fasted controls (Fig. 5B and C). The results demonstrate that PRDX-3 is not essential for the adaptive response to fasting, but both PRDX-2 and PRDX-6 are required for the fasting induced increase in longevity and stress resistance. Furthermore, the effects of fasting on the physiological activity of these strains was assessed by CeleST. The fasting protocol increased travel speed in the wild type strain compared to non-fasted controls on days 6, 10 and 15 and decreased curling on day 15 (Fig. 5D). In contrast fasting had no beneficial effects in the *prdx-2* and *prdx-6* mutant strains. Fasting resulted in decreased activity in the *prdx-2* mutant on days 6 and 10 and increased curling in the *prdx-2* and *prdx-6* mutants on days 10 and 15 (Fig. 5D).

### 2.6 Fasting reduces age-related lipofuscin and lipid accumulation in the wild type but not *prdx-2* or *prdx-6* mutant strains

Accumulation of the auto-fluorescent lipofuscin in the intestines of *C. elegans* is often used as an indicator of biological health and age [38]. There was an age dependent accumulation of autofluorescence in all strains. However, 4 h 5-day fasting resulted in reduced autofluorescence or lipofuscin accumulation, with a significant decrease compared to controls on days 10 and 15 in the wild type strain (Fig. S6A). In the *prdx-2* and *prdx-6* mutants there was increased lipofuscin on days 15 and day 10 respectively compared to non-fasted controls (Fig. S6A). As nutritional stress induces a metabolic shift and mitochondrial remodelling to promote β-oxidation, Oil Red staining was performed to assess levels of neutral triglycerides and lipids in these strains. Similar to the lipofuscin results, there was an age-related increase in Oil Red staining in all strains (Fig. S6B). Moreover, the wild type had decreased lipid accumulation following the fasting protocol on days 6, 10 and 15 compared to controls. However, the *prdx-2* and prdx-6 mutants had increased lipid staining following 4 h 5-day fasting on days 6, 10 and 15 compared to their respective controls (Fig. S6B). Furthermore, following the fasting intervention there was a robust increase in the levels of genes related to mitochondrial β-oxidation (*acs-2*, *acs-11* and *cpt-3*) [5] in the wild type strain, that was not apparent in either the *prdx-2* or *prdx-6* mutant strains (Fig. S6C). The data highlight that fasting reduced age-related lipofuscin and lipid accumulation in the wild type strain, but no beneficial results were obtained in the *prdx-2* and *prdx-6* mutants. The enhanced accumulation of lipofuscin and lipids following fasting in the *prdx-2* and *prdx-6* mutants would suggest disrupted metabolic regulation in these strains.

### 2.7 Fasting promotes redox dependent mitochondrial remodelling that is dependent on PRDX-2 and PRDX-6

**Fig. 6:**
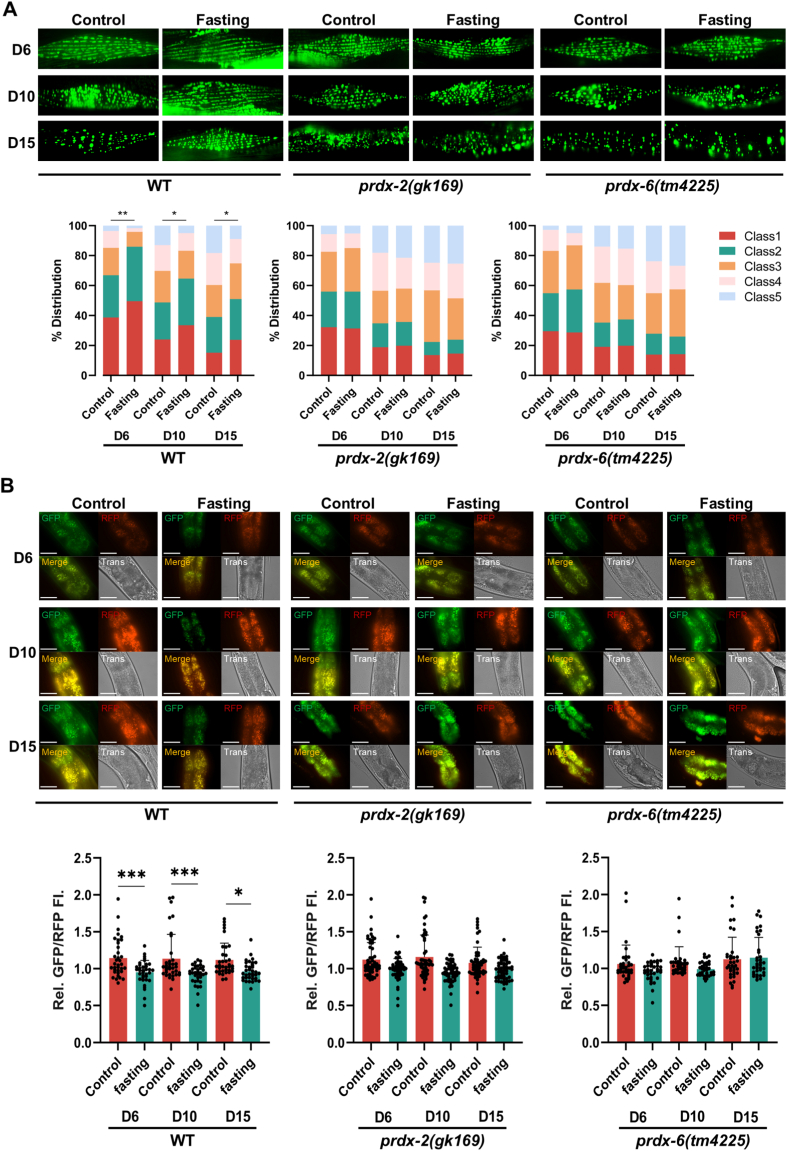
Fasting promotes PRDX-2 and PRDX-6 dependent mitochondrial remodelling and mitophagy during ageing. Representative images of mitochondrial reporter **(A)** and mitophagy reporter **(B)** in the background of wild type, *prdx-2(gk169)*, and *prdx-6(tm4225)* mutants on days 6, 10, and 15 following a 4 h 5-day IF. For mitochondrial morphology, data represent the percentage distribution of 130-150 images per group. ∗p ≤ 0.05 and ∗∗p ≤ 0.01. For mitophagy, data represent the mean ± SEM (n = 35). Scale bars, 50 μm ∗p ≤ 0.05 and ∗∗∗p ≤ 0.001.

To determine the role of mitochondria in the beneficial fasting induced changes, mitochondrial morphology, mitophagy and ROS levels were determined in *prdx-2* and *prdx-6* mutant strains. Consistently, fasting promoted a more elongated mitochondrial network and protected against age-related mitochondrial fragmentation in the control strain (Fig. 6A). The *prdx-2* and *prdx-6* mutants were crossed with the muscle mitochondrial reporter *zcIs14[myo-3p::GFP(mit)]* and mitophagy reporter *unc-119(ed3); Ex[myo-3p::tomm-20::Rosella; unc-119(+)]*. The loss of PRDX-2 and PRDX-6 resulted in increased mitochondrial fragmentation, and fasting had no additional effects on mitochondrial morphology. The fasting protocol resulted in increased mitophagy on day 6, which was maintained on days 10 and 15 in the wild type (Fig. 6B). The mitophagy reporter strain was crossed with both the *prdx-2* and *prdx-6* mutants and no change in mitophagy within muscle determined by the ratio of GFP:RFP was observed as a result of fasting at any of the time points analysed (Fig. 6B).

**Fig. 7:**
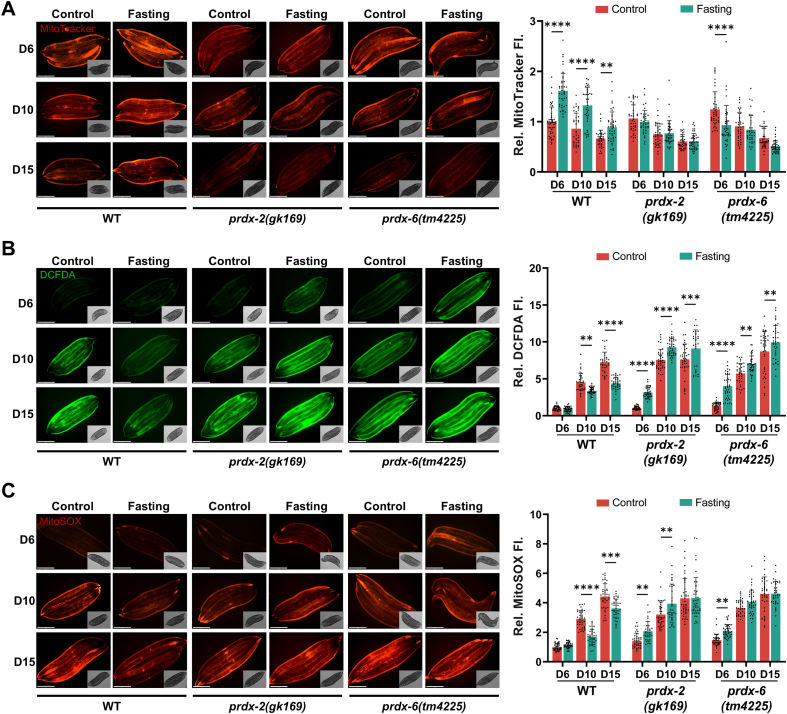
PRDX-2 and PRDX-6 are required for fasting-induced maintenance of mitochondrial membrane potential and to maintain intracellular redox homeostasis. Representative images and quantification of mitochondrial membrane potential assessed by MitoTracker Red staining (**A**), intracellular total ROS assessed by DCFDA staining (**B**), and mitochondrial ROS assessed by MitoSOX staining (**C**) in wild type N2, *prdx-2(gk169)*, and *prdx-6(tm4225)* strains on days 6, 10, and 15 following 4 h 5-day intermittent fasting. Scale bars, 275 μm ∗∗p ≤ 0.01, ∗∗∗p ≤ 0.001, ∗∗∗∗p ≤ 0.0001 (n = 35-45).

The effects of fasting on mitochondrial membrane potential were estimated using MitoTracker Red CMXRos staining, which revealed an age dependent decrease in mitochondrial membrane potential (Fig. 7A). However, there was an increase in MitoTracker Red CMXRos staining in the wild type strain compared to non-fasted controls on days 6, 10 and 15, suggesting increased mitochondrial membrane potential as a result of the 5-day fasting intervention (Fig. 7A). Similarly, increased TMRM staining was also observed in adult day 6 wild type worms following the 5 day fasting protocol (Fig. S7). No changes in MitoTracker Red CMXRos staining were observed following fasting in the *prdx-2* and *prdx-6* mutants. To assess changes in the redox environment following the fasting protocol, DCFDA and MitoSOX staining was performed. Following the early life fasting protocol there was decreased DCFDA and MitoSOX staining on days 10 and 15 in the wild type strain compared to non-fasted controls but the opposite occurred in both the *prdx-2* and *prdx-6* mutants, with increased staining on days 6 and 10 compared to non-fasted controls indicating a more oxidative redox environment (Fig. 7B and C). Together these results highlight that fasting helped prevent age-related mitochondrial fragmentation, maintained by increased mitophagy and mitochondrial membrane potential with a decreased oxidative environment in wild type fasted worms. However, strains lacking PRDX-2 and PRDX-6 had increased mitochondrial fragmentation and an increased oxidative intracellular environment with age following fasting.

### 2.8 PRDX-2 and PRDX-6 are required for SKN-1 activation and DAF-16 nuclear localisation following fasting

**Fig. 8:**
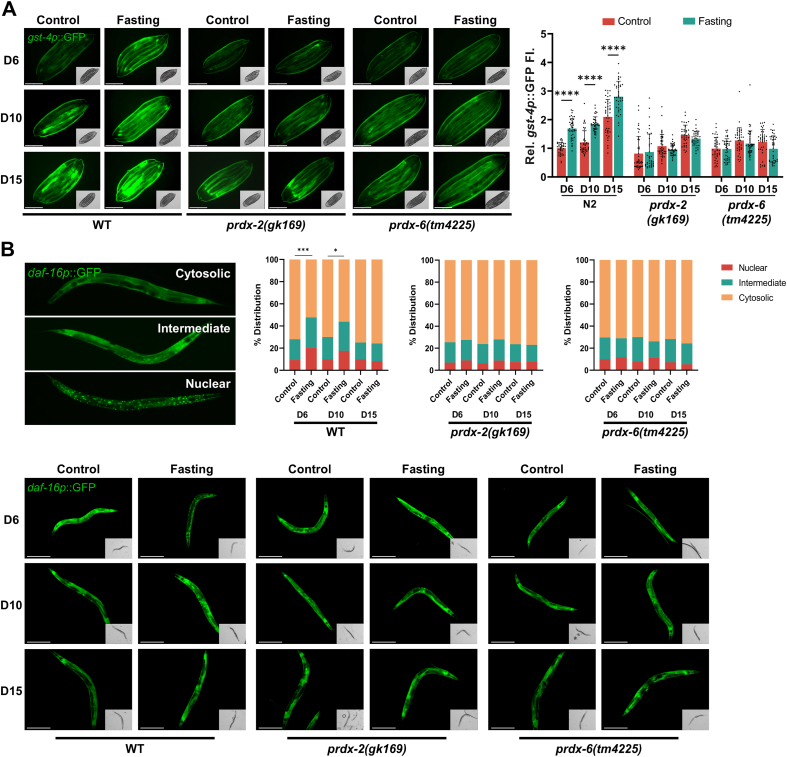
PRDX-2 and PRDX-6 are required for SKN-1 activation and DAF-16 nuclear localisation following fasting. (A) Representative images of SKN-1 activation reporter *dvIs19[(pAF15)gst-4p::GFP::NLS] III* under wild-type N2, *prdx-2(gk169)*, and *prdx-6(tm4225)* mutant backgrounds on days 6, 10, and 15 following a 4 h 5-day IF. Scale bars: 275 μm ∗∗∗∗p ≤ 0.0001 (n = 37-44). **(B)** Representative images of DAF-16 nuclear localisation assessed using the *zIs356[daf-16p::daf-16a/b::GFP + rol-6(su1006)]* reporter strain under the wild type, *prdx-2(gk169)*, and *prdx-6(tm4225)* mutant backgrounds on days 6, 10, and 15 following the 4 h 5-day IF. Images were categorised as: cytosolic, intermediate, and nuclear localisation. Data represent the percentage distribution of 40 animals per group. ∗p ≤ 0.05 and ∗∗∗p ≤ 0.001.

As demonstrated previously SKN-1 was activated following the fasting intervention (Fig. 3A). The 4 h 5-day fasting intervention maintained elevated SKN-1 activation on days 6, 10 and 15 compared to non-fasted controls (Fig. 8A). The SKN-1 reporter *dvIs19[(pAF15)gst-4p::GFP::NLS] III* was crossed with the *prdx-2* and *prdx-6* mutant strains and no activation of SKN-1 was observed following fasting, despite the elevated oxidative environment in these strains (Fig. 8A). DAF-16 has repeatedly been demonstrated to be one of the key regulators of nutritional stress and its nuclear localisation activates pathways related to autophagy [39]. The DAF-16 nuclear localisation was assessed on days 6, 10 and 15 using the *zIs356[daf-16p::daf-16a/b::GFP + rol-6(su1006)]* reporter strain. The 5-day fasting protocol increased DAF-16 nuclear localisation on day 6, that was maintained on day 10 but returned to baseline on day 15 (Fig. 8B). The DAF-16 reporter was also crossed with both the *prdx-2* and *prdx-6* mutant strains and no change in nuclear localisation was observed at any timepoint (Fig. 8B). The results highlight that the 4 h 5-day fasting protocol promoted prolonged SKN-1 activation and DAF-16 nuclear localisation that was not evident in strains lacking either PRDX-2 or PRDX-6.

### 2.9 PRDX-2 and PRDX-6 are required for increased *daf-16* and *hlh-30* expression following fasting

**Fig. 9:**
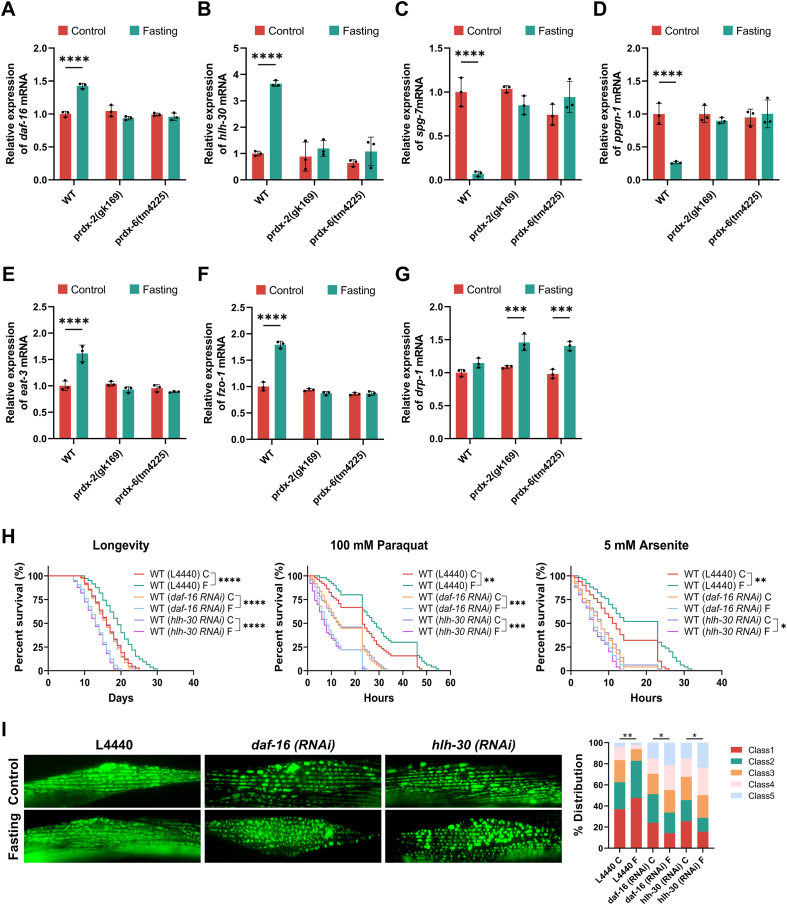
PRDX-2 and PRDX-6 are required for fasting-induced *daf-16* and *hlh-30* expression and subsequent adaptive mitochondrial remodelling. (A-G) Relative mRNA expression levels of the transcription factors *daf-16***(A)** and *hlh-30***(B**), the mitochondrial proteases *spg-7***(C)** and *ppgn-1***(D**), the mitochondrial fusion genes *eat-3***(E)** and *fzo-1***(F**), and the mitochondrial fission gene *drp-1***(G)**. Expression levels were analysed by qPCR in wild-type N2, *prdx-2(gk169)*, and *prdx-6(tm4225)* mutant strains at adult day 6 under control and 4 h 5-day fasting conditions. ∗∗∗p ≤ 0.001 and ∗∗∗∗p ≤ 0.0001 (n = 3). **(H)** Survival assays evaluating longevity, mitochondrial oxidative stress resistance induced by 100 mM paraquat, and cytoplasmic oxidative stress resistance induced by 5 mM sodium arsenite following 4 h 5-day fasting. Assays were performed at adult day 6 in wild type animals treated with empty vector (L4440), *daf-16 (RNAi)*, or *hlh-30 (RNAi)* under control and fasting conditions. Kaplan–Meier survival plots represent experiments initiated with 105 animals per group. ∗∗p ≤ 0.01, ∗∗∗p ≤ 0.001 and ∗∗∗∗p ≤ 0.0001. **(I)** Representative images of mitochondrial morphology within body wall muscle of *zcIs14[myo-3p::GFP(mit)*] strain treated with empty vector (L4440), *daf-16 (RNAi)*, or *hlh-30 (RNAi)* under control and fasting conditions. Data represent the percentage distribution of 130-150 images per group. ∗p ≤ 0.05 and ∗∗p ≤ 0.01.

DAF-16 and HLH-30 transcription factors are key regulators of autophagy and the response to nutritional stress; they can act synergistically to link both autophagy and lipolysis [19]. Furthermore, both DAF-16 and HLH-30 activity or nuclear localisation is linked to their phosphorylation and sequestration in the cytoplasm. However, there is also evidence that their nuclear translocation is dependent on redox signalling [18,34]. The expression levels of both *daf-16* and *hlh-30* increased following 4 h fasting over 5 days in the wild type strain but not in the *prdx-2* or *prdx-6* mutants (Fig. 9A and B). DAF-16 nuclear localisation results in the negative inhibition of the mitochondrial protease genes *spg-7* and *ppgn-1*, that degrade the inner mitochondrial fusion protein EAT-3 [8]. Following fasting there were decreased expression levels of both *spg-7* and *ppgn-1* and increased levels of mitochondrial fusion genes *eat-3* and *fzo-1* in the wild type strain compared to non-fasted controls, that was not observed in the *prdx-2* or *prdx-6* mutants (Fig. 9C–F). Interestingly, there were increased expression levels of the mitochondrial fission gene *drp-1* in both *prdx-2* and *prdx-6* mutant strains following fasting (Fig. 9G). As there was a blunted increase in the mRNA expression levels of *daf-16* and *hlh-30* in the *prdx-2* and *prdx-6* mutants, RNAi was used to target both *daf-16* and *hlh-30* to determine their regulatory role in the response to fasting. Knockdown of either *daf-16* or *hlh-30*, resulted in decreased longevity and stress resistance to both paraquat and sodium arsenite in response to fasting (Fig. 9H). Consistently, *daf-16* or *hlh-30* RNAi administration resulted in compromised mitochondrial morphology within body wall muscle cells following fasting (Fig. 9I). These results highlight that fasting can induce the expression of *daf-16* and *hlh-30* and their activities are essential for the adaptive response to nutritional stress promoting mitochondrial fusion, and this response required the presence of PRDX-2 and PRDX-6.

**Fig. 10:**
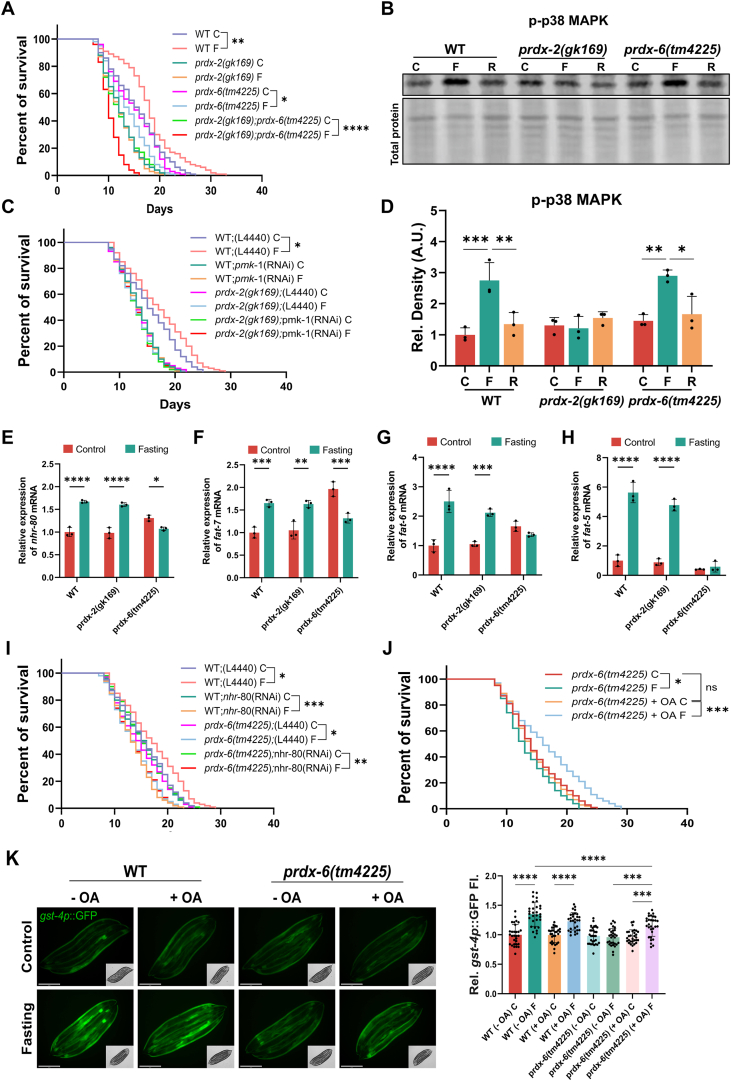
PRDX-2 and PRDX-6 mediate the fasting-induced adaptive response through distinct p38 MAPK and lipid signalling pathways. (A) Lifespan of wild type N2, *prdx-2(gk169)*, *prdx-6(tm4225)*, and the double *prdx-2; prdx-6* mutant strains subjected to the 4 h 5-day IF. Kaplan–Meier survival plots represent experiments initiated with 105 animals per group. ∗p ≤ 0.05, ∗∗p ≤ 0.01, and ∗∗∗∗p ≤ 0.0001. **(B, D)** Western blot images and quantification of phosphorylated p38 MAPK in day 1 adult wild type, *prdx-2(gk169)*, and *prdx-6(tm4225)* strains under control (C), fasting (F), and recovery (R, 16 h) conditions. ∗p ≤ 0.05, ∗∗p ≤ 0.01, and ∗∗∗p ≤ 0.001. **(C)** Lifespan of wild type N2 and *prdx-2(gk169)* strains treated with empty vector (L4440) or *pmk-1 (RNAi)* under control and 4 h 5-day IF conditions. Kaplan–Meier survival plots represent experiments of 105 animals per group. ∗∗p ≤ 0.01. **(E**–**H)** Relative mRNA expression levels of the lipid metabolism transcriptional regulator *nhr-80* and the fatty acid desaturases *fat-7*, *fat-6*, and *fat-5* in day 6 adult wild type, *prdx-2(gk169)*, and *prdx-6(tm4225)* strains under control and 4 h 5-day IF conditions. ∗p ≤ 0.05, ∗∗p ≤ 0.01, ∗∗∗p ≤ 0.001, and ∗∗∗∗p ≤ 0.0001 (n = 3). **(I)** Lifespan of wild type N2 and *prdx-6(tm4225)* strains treated with empty vector (L4440) or *nhr-80 (RNAi)* under control and 4 h 5-day IF conditions. Kaplan–Meier survival plots represent experiments initiated with 105 animals per group. ∗p ≤ 0.05, ∗∗p ≤ 0.01, and ∗∗∗p ≤ 0.001. **(J)** Lifespan assay evaluating the effect of oleic acid (OA) supplementation on the day 6 adult *prdx-6(tm4225)* mutant strain under control and 4 h 5-day IF conditions. Kaplan–Meier survival plots represent experiments initiated with 105 animals per group. ∗p ≤ 0.05, and ∗∗∗p ≤ 0.001. **(K)** Representative images of SKN-1 reporter in wild type and *prdx-6(tm4225)* mutant backgrounds treated with or without 0.8 mM of OA following the 4 h 5-day IF. Images were acquired from day 6 adult worms. (Scale bars: 275 μm). ∗∗∗p ≤ 0.001, and ∗∗∗∗p ≤ 0.0001 (n = 30).

In order to dissect the roles of PRDX-2 and PRDX-6 in the response to fasting an epistasis approach was taken, and a double *prdx-2*;*prdx-6* mutant strain was assessed. The double *prdx-2*;*prdx-6* mutant had decreased longevity following fasting compared to *prdx-2* and *prdx-6* mutants suggesting that they function via distinct cellular signalling pathways (Fig. 10A). It has previously been demonstrated that PRDX-2 was involved in the activation of the p38 MAPK PMK-1 signalling pathway [37]. Following fasting there was an increase in phospho-p38 in the wild type and the *prdx-6* mutant strains, but no increase in the *prdx-2* mutant (Fig. 10B–D). Moreover, RNAi knockdown of the *pmk-1* in both wild type and the *prdx-2* mutant failed to result in a beneficial adaptive response to fasting (Fig. 10C). Similarly, incubation of the wild type strain with NAC during fasting did not result in an increase in phospho-p38 (Fig. S8A). These results suggest that PRDX-2 is required for p38 MAPK PMK-1 activation in response to elevated ROS during fasting.

PRDX-6 is a multifunctional enzyme that has a key role in lipid signalling and prevention of ferroptosis in mammalian models [30]. Thus, a number of key genes involved in regulating lipid signalling were quantified in the different strains. In both the wild type and *prdx-2* mutant strains, following fasting there was a robust increase in the levels of *nhr-80* (Fig. 10E), a transcriptional regulator of lipid metabolism that controls the expression of fatty acid desaturases in response to cellular stress [40]. The increase in *nhr-80* was accompanied by an increase in the expression levels of *fat-5*, *fat-6* and *fat-7* in response to fasting in both wild type and *prdx-2* mutants (Fig. 10F–H). However, there was no increase in the levels of *nhr-80*, *fat-5, fat-6* or *fat-7* in the *prdx-6* mutant following fasting, although there was basally elevated expression level of *fat-6* and *fat-7* in the non-fasted *prdx-6* mutant (Fig. 10E–H). No changes were detected following fasting in the levels of the lipid regulators *nhr-49*, *sbp-1* or *mdt-15* in any of the strains (Fig. S8B). Incubation of the wild type strain with NAC also failed to increase the expression levels of *nhr-80*, *fat-5*, *fat-6* or *fat-7* following the fasting protocol (Fig. S8C). Moreover, RNAi knockdown of the *nhr-80* in both wild type and the *prdx-6* mutant strains resulted in a decreased longevity response to fasting (Fig. 10I). Notably, *nhr-80* RNAi did not further diminish the lifespan of the *prdx-6* mutant compared to the wild type strain following the fasting regimen (Fig. 10I). Given that fatty acid desaturases (FAT-5, FAT-6 and FAT-7) are involved in the generation of monounsaturated fatty acids such as oleic acid (OA) [41], we further investigated whether OA supplementation could rescue the *prdx-6* mutant in response to fasting. The longevity data indicate that supplementation with OA in the *prdx-6* mutant undergoing fasting extended its lifespan (Fig. 10J). Fasting increased SKN-1 activation in the wild type strain that was not apparent in the *prdx-6* mutant, however inclusion of OA partially rescued this response (Fig. 10K). Similarly, fasting increased DAF-16 nuclear localisation in the wild type strain, which was not observed in the *prdx-6* mutant. However, OA was not able to rescue the increased DAF-16 nuclear localisation following fasting in the *prdx-6* mutant (Fig. S9A). Furthermore, RNAi mediated knockdown of *nhr-80* attenuated the fasting induced activation of SKN-1 in wild-type *C. elegans*, whereas OA supplementation fully restored this activation (Fig. S9B). The results highlight that under fasting conditions PRDX-6 is required for induction of the lipid regulator NHR-80 and OA supplementation can partially rescue the altered lipid remodelling in this mutant.

Together the data presented in this manuscript demonstrates that nutritional stress induced by fasting results in transient endogenous ROS generation accompanied by extensive metabolic and lipid reprogramming, highlighted by mitochondrial remodelling and ultimately lifespan extension. The data highlight that two highly conserved Peroxiredoxins, PRDX-2 and PRDX-6, are required for this adaptive response, albeit via different metabolic pathways. PRDX-2 was required for the activation of p38 MAPK PMK-1 and appropriate stress response, while PRDX-6 was required for the lipid remodelling in response to fasting, but both pathways ultimately converged on SKN-1 and DAF-16 signalling.

## 3 Discussion

During an early life fasting intervention to induce nutritional stress in *C. elegans*, there was an increase in endogenous ROS generation that returned to baseline following a recovery period. The fasting intervention resulted in dynamic mitochondrial remodelling which ultimately increased longevity and helped prevent an age-related decline in activity. Treatment of worms with the antioxidant NAC has previously been demonstrated to prevent the increased localised ROS generation and longevity under conditions of glucose restriction [15]. An increase in mitochondrial superoxide/H_2_O_2_ during fasting and metabolic stress has previously been observed during a shift in metabolism towards increased β-oxidation [42,43]. A switch in the metabolic fuel source to increased fatty acid oxidation has been associated with a more fragmented mitochondrial morphology in mammalian cells [44]. In this study, elongation of the mitochondrial network was observed following a recovery period in the wild type. The fasting protocol also increased SKN-1 activity and DAF-16 nuclear localisation, which were required for the beneficial adaptive response. However, fasting did not result in any beneficial adaptations in mutant strains lacking the 2-Cys PRDX-2 or the 1-Cys PRDX-6. These mutant strains displayed a distinct fragmented muscle mitochondrial morphology. During the fasting protocol, levels of ROS were increased in all strains but remained elevated following a recovery period in the *prdx-2* and *prdx-6* mutant strains, indicating a disrupted intracellular redox environment. An epistasis approach demonstrated that the double *prdx-2*;*prdx-6* mutant strain had an additive effect, indicating that PRDX-2 and PRDX-6 respond to acute elevated ROS levels via distinct signalling pathways.

Neither the *prdx-2* nor *prdx-6* mutant strains transiently increased SKN-1 activity or DAF-16 nuclear localisation following the fasting protocol. Under most conditions of oxidative stress, p38 PMK-1 kinase phosphorylates SKN-1 which is required for its nuclear accumulation [22]. The data presented here indicates that mutants lacking PRDX-2 did not activate the p38 PMK-1 pathway following the fasting protocol that was required for the adaptive response. In contrast, the *prdx-6* mutant did not activate a lipid remodelling response following the shift in the metabolic fuel source during fasting. The fasting protocol generated a robust elevation in expression levels of *nhr-80* and its target genes, *fat-5*, *fat-6* and *fat-7*, in both the wild type and the *prdx-2* mutant, which was not apparent in the *prdx-6* mutant. As FAT-5, FAT-6 and FAT-7 are fatty acid desaturases involved in the generation of monounsaturated fatty acids (MUFAs), the supplementation of OA in the *prdx-6* mutant was able to extend the lifespan of this strain and partially rescued SKN-1 activation following fasting. Together, the results indicate that the initial transient increase in ROS during fasting is a key signalling response that requires PRDX-2 to activate the p38 PMK-1 signalling pathway and PRDX-6 to activate lipid remodelling.

Peroxiredoxins are highly abundant proteins that constitute up to ∼1% of total cellular protein and generally considered as antioxidant peroxidases [45]. The peroxiredoxin family exhibit metazoan wide conservation, and 1-Cys peroxiredoxin such as PRDX-6 is the most highly conserved peroxiredoxin family member of the 18 species analysed [23]. The highly conserved nature of these proteins would suggest that they have more functions than merely as antioxidant peroxidases but have key roles in transmitting the transient endogenous increase in intracellular ROS in a redox signalling cascade. In *C. elegans*, PRDX-2 has previously been demonstrated to be required for redox dependent light sensing in sensory neurons [46], while also required for the lifespan extension when worms are grown at 15 °C and when treated with metformin [27,47]. PRDX-2 is also required for the beneficial redox dependent adaptive response to exercise [28]. Exercise promotes an increase in endogenous ROS generation and the *prdx-2* mutant strain had increased reversible oxidation of specific Cys residues and reduced lifespan following exercise [28]. Furthermore, the *prdx-2* mutant strain had disrupted DAF-16 nuclear localisation in response to exercise, a more fragmented mitochondrial morphology with disrupted mitochondrial ER contact sites [7]. However, it has been reported that the *prdx-2* mutant at L2/L3 larval stages has increased DAF-16 and SKN-1 activities [37]. In contrast, in this study adult strains lacking PRDX-2 analysed at day 6 following the 5-day fasting protocol, the *prdx-2* mutant had impaired DAF-16 and SKN-1 activation, which may be due to the accelerated ageing of this strain. Interestingly it has also been reported that hyperoxidation of peroxiredoxins disrupt cell signalling pathways with a reduced redox stress response capacity associated with senescence [31]. Increased peroxiredoxin hyperoxidation has also been reported with age in *C. elegans* [7].

The 1-Cys peroxiredoxins are the most highly conserved members of the peroxiredoxin family [23], in mammals this multifunctional enzyme has been increasingly connected to the repair of oxidised phospholipids and protection against ferroptosis induced cell death [30,48,49]. In *C. elegans*, a *prdx-6* mutant strain has recently been shown to have elevated lipid oxidation and was more susceptible to cell death when treated with diethyl maleate, which depletes glutathione levels [50]. The results presented in this study demonstrate that in the wild type and *prdx-2* mutant fasting induced a strong elevation in expression levels of *nhr-80* and the expression of downstream fatty acid desaturases *fat-5*, *fat-6* and *fat-7* [40], but this response was not observed in the *prdx-6* mutant. However, supplementation with OA in the *prdx-6* mutant partially rescued this phenotype. The increased expression of *nhr-80* but not *nhr-49* under the fasting conditions performed here is interesting and warrants further investigation. NHR-49 is considered a key regulator of fatty acid desaturase and mitochondrial β-oxidation genes [51], while NHR-80 regulates fatty acid desaturase genes [41]. An epistasis approach has suggested that both NHR-49 and NHR-80 lie in the same pathway to regulate longevity in response to hypoxia and oxidative stress, and that NHR-80 can interact with NHR-49 under certain conditions [52]. It has also been recently demonstrated that *prdx-6* mutants have elevated NHR-49 dependent *fmo-2* expression that was responsible for the increased stress resistance of these mutants at L4 stage [50]. Together this would suggest that lipid remodelling in response to metabolic, hypoxia and oxidative stress is dependent both on age and the type and severity of stress.

The *prdx-2* mutant strain had disrupted p38 PMK-1 signalling and the *prdx-6* mutant failed to activate lipid remodelling in response to the fasting protocol, yet both mutant strains had altered DAF-16 and SKN-1 activities and disrupted mitochondrial morphologies. There are clear links between the activities of both DAF-16 and SKN-1 in response to redox stress but also in the regulation of lipid homeostasis and mitochondrial dynamics. DAF-16 regulates many key genes associated with fat metabolism [53]. The long lived *daf-2* mutant requires DAF-16 for lipid or fat storage when IIS pathways were reduced [54]. Treatment of *C. elegans* with the redox cycler paraquat to increase intracellular ROS, can inhibit insulin signalling and result in the subsequent activation of DAF-16, which stimulated *fat-5* expression and resulted in fat accumulation [55]. However, DAF-16 activation is not required for the lifespan extension as a result of chronic caloric restriction in studies using *eat-2* mutant strains, but essential for lifespan extension induced by intermittent fasting or following caloric restriction in middle aged worms [56,57]. Reduced IIS pathways also resulted in increased nuclear accumulation of SKN-1 that promotes longevity independently from DAF-16 [12]. Transient impairment of IIS by decreasing *daf-2* expression can acutely increase ROS levels, promote mitochondrial metabolism and extend longevity with ROS levels decreasing following the adaptive response [58]. However, SKN-1 was required for this response following the acute increase in ROS generation during reduced IIS signalling [58]. Similarly, inhibition of mitochondrial Complex I and dietary restriction results in an increase in ROS that activates p38 PMK-1 and SKN-1 and lifespan extension [59]. Moreover, SKN-1 regulates genes in the adaptive response to starvation and constitutive activation resulted in a starvation response [10]. SKN-1 has been demonstrated to coordinate mitochondrial dynamics by regulating the expression of mitochondrial biogenesis genes and mitophagy genes such as *dct-1*, the ortholog of mammalian mitophagy receptor BNIP3 and NIX/BNIP3L [13]. SKN-1 has also been reported to regulate genes involved in lipid metabolism and promotion of fatty acid oxidation under starvation conditions [11]. In long lived worms that are lacking the germ cell line GSC(−), these strains have altered lipid metabolism as they are unable to reproduce but continue to make lipid rich yolk [60]. The build-up of lipids in GSC(−) worms has been demonstrated to activate SKN-1, which inhibits further lipid accumulation, while the reduction of SKN-1 in GSC(−) strains results in higher lipid levels [60]. The results presented in this study highlight that dual activation of both DAF-16 and SKN-1 following the fasting protocol resulted in an adaptive stress response that was dependent on PRDX-2 and PRDX-6, resulting in mitochondrial and lipid remodelling.

Tissue specific metabolic remodelling occurs in response to a variety of genetic and physiological stressors. A limitation of this study was that mitochondrial morphology and mitophagy was assessed using muscle specific reporters but many of the immediate metabolic responses to fasting are intestinal. A more accurate assessment of the immediate effects of fasting would be to include intestinal and neuronal specific reporters of mitophagy. The fasting induced changes in overall mitochondrial content were assessed using the muscle mitochondrial reporter *zcIs14[myo-3p::GFP(mit)]* at days 6, 10 and 15. These results highlight the age-related changes in muscle mitochondrial content with no obvious changes in mutants lacking PRDX-2 or PRDX-6. However, it may not reflect changes in mitochondrial content within other tissues such as the intestine or nervous systems. Furthermore, the 5-day fasting protocols were initiated on adult day 1 worms for either 4, 8 or 12 h, with experimental analysis performed on days, 6, 10 and 15. Although every effort was made to ensure that the refeeding times were consistent across groups this may have had an effect on any data presented for day 6, where there were slight differences in the refeeding times. Similarly, the longevity assays performed following the fasting protocol were assessed using NGM plates containing FUdR, which enable synchronised lifespan assays by preventing progeny development. However, FUdR is not inert and has been previously reported to result in lifespan extension in mitochondrial mutants, activation of DAF-16 and proteostasis [61,62]. As FUdR can affect longevity-associated pathways, the results presented and conclusions reached are restricted to relative differences under identical experimental conditions. The bleaching synchronisation approach used in this study results in a period of starvation-induced L1 arrest accompanied with suppression of IIS and activation of DAF-16, as a result the population of worms have a shared early-life metabolic stress compared to alternative synchronisation approaches where populations of worms have access to food from baseline.

The results presented highlight the role of PRDX-6 in coordinating lipid remodelling. The mammalian ortholog of PRDX-6 has recently been reported as critically important in preventing ferroptosis induced cell death as a result of lipid peroxidation [30,48]. Furthermore, loss of Prdx6 in mammalian models results in altered lipid homeostasis, increased lipid peroxidation and susceptibility to ferroptosis [30]. Similarly in *C. elegans* strains lacking PRDX-6, the strain has increased sensitivity to toxicity as a result of glutathione depletion following treatment with diethyl maleate [50]. Our data highlight that loss of PRDX-6 results in a failure to activate lipid remodelling dependent on *nhr-80* assessed by gene expression. Analysis of protein levels of NHR-80 and including tissue specific lipidomic analysis would significantly strengthen the results obtained and confirm the essential role of PRDX-6 in coordinating lipid remodelling.

In summary, dietary restriction induced by a fasting protocol extended longevity and stress resistance in the wild type strain. The fasting protocol induced an acute increase in intracellular ROS, activation of DAF-16 and SKN-1 transcription factors resulting in mitochondrial remodelling. The early life fasting protocol helped maintain mitochondrial morphology and physiological activity with age. These adaptive responses were dependent on both PRDX-2 and PRDX-6, albeit via distinct molecular signalling pathways. The loss of PRDX-2 resulted in a failure to activate p38 PMK-1 and the adaptive oxidative stress response via SKN-1 following fasting but also resulted in decreased DAF-16 nuclear localisation. While the failure of the *prdx-6* mutant strain to adapt to fasting was a result of altered lipid remodelling and subsequent disrupted activation of both DAF-16 and SKN-1. Together these results highlight that endogenous ROS generation, as a result of nutritional stress, activates distinct metabolic pathways that are sensed by the highly conserved peroxiredoxins, PRDX-2 and PRDX-6. Ultimately the signalling pathways activated following fasting converged on DAF-16 and SKN-1 dependent stress response pathways. The subsequent mitochondrial remodelling helped promote physiological activity and longevity. The results highlight a strong interconnected feedback loop between metabolism, redox signalling and lipid remodelling that requires PRDX-2 and PRDX-6 to sense alterations in the intracellular redox environment.

## 4 Materials and methods

### 4.1 *C. elegans* strains and maintenance

*C. elegans* were cultured on nematode growth medium (NGM) plates seeded with *Escherichia coli* (*E. coli*) OP50 and maintained in the dark at 20 °C. Strains used in this study are listed in Reagents and Resources table.

### 4.2 *C. elegans* intermittent fasting

All experiments were performed by synchronisation of worms using a standard bleaching protocol that allows eggs to hatch overnight (12-16 h) in M9 buffer resulting in L1 arrest under food deprived conditions. The L1 larvae were then transferred to NGM plates and grown to day 1 (D1) adults. Synchronised D1 adults were transferred to either fresh NGM plates seeded with OP50 (Control) or unseeded NGM plates (Fasting group). For the fasting group, nematodes were starved for 4, 8, or 12 h before being refed by transferring them back to fresh OP50-seeded NGM plates. A single fasting event on day 1 of adults was defined as “single fasting,” whereas five fasting events from day 1 to day 5 of adults were defined as “5-day intermittent fasting”. Oleic acid supplementation plates were made using an adapted protocol [63,64]. In brief, 100 mM aqueous solution stocks of sodium oleate (Sigma) were made fresh before pouring regular NGM plates containing 0.001% Tergitol (NP40) and added to NGM media once cooled to approximately 55 °C to a final concentration of 0.8 mM. Plates containing NAC were prepared as described previously [15]. Briefly, a 0.5 M aqueous stock was made fresh before pouring regular NGM plates and added to NGM media once cooled to approximately 55 °C to a final concentration of 5 mM.

### 4.3 Lifespan assays

Lifespan assays were initiated following the final day of single fasting or 5-day intermittent fasting. A total of 105 nematodes were randomly distributed across three seeded NGM plates supplemented with 10 μM 5-fluoro-2′-deoxyuridine (FUdR) to inhibit egg hatching. The number of live and dead nematodes was recorded every one to two days until all individuals died. Nematodes that crawled off the plates or burrowed into the agar were censored.

### 4.4 Oxidative stress survival assays

Paraquat and sodium arsenite were utilised to induce mitochondrial and cytoplasmic oxidative stress, respectively. Fresh aliquots of 250 μL of 100 mM paraquat or 5 mM sodium arsenite, diluted in M9 buffer, were dispensed into each well of a 24-well plate. Subsequently, 12 to 14 nematodes, totalling 50 nematodes per group, were randomly placed into each well. The number of surviving nematodes was recorded every 1-2 h until all individuals died. Nematodes were considered dead if they failed to respond to gentle stimulation with a platinum wire picker [65].

### 4.5 *C. elegans* swimming test (CeleST)

To evaluate swimming capacity, the CeleST assay was conducted as described previously [33]. Briefly, five nematodes were randomly selected and placed into a 40 μL droplet of M9 buffer, which were confined within a 10 mm circular barrier on a glass slide. Nematodes were allowed to settle in the M9 buffer for 20 s, then their movement was recorded for 30 s at 15 frames per second using a Nikon LV-TV microscope equipped with an OPTIKA C–P20CM camera at 1x magnification. Data processing and the determination of swimming metrics were performed using a custom MATLAB application. A minimum of 30 nematodes per condition were evaluated.

### 4.6 Quantitative real-time PCR (qPCR)

Total RNA was extracted from the nematodes using the standard TRIzol reagent method [66]. For cDNA synthesis, 500 ng of total RNA and 1 μL of random hexamers were co-incubated at 65 °C for 10 min. Subsequently, 4 μL of RT buffer, 2 μL of DTT, 1 μL of RiboLock RNase Inhibitor, 1 μL of dNTP mix, and 1 μL of SuperScript II Reverse Transcriptase were added, and the reaction was incubated at 42 °C for 1 h. Real-time quantitative PCR was performed using Fast SYBR Green Master Mix, with *cdc-42* serving as the housekeeping gene. The sequences of primers used in this study are listed in Table S1.

### 4.7 Western blotting

Total nematode proteins were extracted using a lysis buffer (150 mM NaCl, 20 mM Tris pH 7.5, 1 mM EDTA pH 8.3, 0.5% SDS, 1% Triton, 50 mM NEM) supplemented with 1% protease inhibitor cocktail and 10% phosphatase inhibitor cocktail, followed by protein quantification using the Bradford assay. Equal volumes of protein extracts from each group were loaded on 12% reducing or non-reducing sodium dodecyl sulphate-polyacrylamide gel electrophoresis (SDS-PAGE) gels and subsequently transferred to nitrocellulose membranes. The entire membrane was stained with Ponceau S to ensure equal loading and for normalisation purposes. After a 10-min wash with TBS-T, the membranes were blocked in 5% non-fat milk or 5% BSA in TBS-T for 1 h at room temperature. Following blocking, the membranes were washed three times with TBS-T and incubated with primary antibodies overnight at 4 °C. The membranes were then washed three additional times with TBS-T and incubated with secondary antibodies at a dilution of 1:15,000 in TBS-T for 1 h at room temperature in the dark. Protein bands were detected using the Odyssey Fc imaging system (LI-COR Biosciences). Subsequent densitometric quantification and normalisation of the blots were performed using Image Studio Lite 5.2 software. Uncropped Western blot images are shown in Fig. S10.

### 4.8 RNA interference (RNAi)

RNAi was performed using the feeding method, wherein nematodes were fed *E. coli* HT115 engineered to express double-stranded RNA (dsRNA) targeting specific genes. Individual bacterial colonies were inoculated into Luria-Bertani (LB) broth containing 100 μg/mL ampicillin and incubated overnight at 37 °C with shaking to amplify the cultures. Next, NGM plates supplemented with 1 mM isopropyl β-d-1-thiogalactopyranoside (IPTG) and 100 μg/mL ampicillin were seeded with 100 μL aliquots of the RNAi bacterial culture and incubated at 37 °C for 48 h to induce dsRNA expression. Age-synchronised L1 larvae were cultivated on these RNAi plates until they reached adulthood. The offspring generated from these adults on the RNAi plates were subsequently used for the intermittent fasting experiments. The *E. coli* HT115 with pL4440 empty vector was used as a negative control. qPCR was conducted to assess the knockdown efficiency of the target genes following RNAi treatment (Fig. S11).

### 4.9 *C. elegans* imaging

All images were acquired using the EVOS M7000 imaging system. For fluorescence imaging, identical microscope settings, including illumination intensity, magnification, and channel settings, were applied across comparable groups within each experiment. Quantification was performed using the original raw images without contrast enhancement. Fluorescence intensity was measured using ImageJ. Briefly, the region of interest corresponding to each worm was manually selected, and the area, mean fluorescence intensity, and integrated density were measured. Background fluorescence was measured from adjacent regions without worms or obvious fluorescent signal within the same image. Corrected total cell fluorescence (CTCF) was calculated as follows: CTCF = integrated density − area of selected worm × mean fluorescence of background [67]. The same measurement parameters were applied consistently to all images within each experiment.

For mitochondrial staining, nematodes were incubated with 2.5 μM MitoTracker Red CMXRos for 10 min or 10 μM MitoSOX Red for 1 h. To assess the intracellular ROS, nematodes were soaked in 25 μM H2DCFDA for 1 h. Following incubation, worms were transferred to seeded NGM plates and allowed to feed for 2 h to clear unabsorbed dyes from the gut. Subsequently, the nematodes were immobilised on unseeded NGM plates using a drop of 40 mM levamisole, and images were acquired at 10× magnification. All staining and imaging procedures were performed in the dark. Images of 30 to 45 nematodes from each group were captured and quantified using ImageJ.

For body size measurements following the 5-day intermittent fasting protocol, 30 nematodes per group were paralyzed on unseeded NGM plates and imaged under bright-field microscopy on days 6, 10, and 15 of adulthood. Similarly, to quantify lipofuscin accumulation, worms were directly immobilised with 40 mM levamisole on 4% agarose pads, and intestinal lipofuscin autofluorescence was captured using the DAPI channel at 10× magnification.

Oil Red O staining was performed as previously described with minor modifications [68]. Briefly, collected nematodes were washed with M9 buffer containing 0.01% Tween 20, fixed in 1% paraformaldehyde for 15 min, and subjected to three consecutive freeze-thaw cycles. The worms were then stained in a filtered 60% Oil Red O working solution for 2 h. After staining, nematodes were washed three times with M9 buffer containing 0.01% Tween 20 and mounted on 4% agarose pads for colour image acquisition. Colour images were then quantified by determining the excess red intensity, calculated by subtracting the average intensity of the blue and green channels from the red channel intensity, as described previously [69].

Mitochondrial content and morphology in body wall muscle cells were monitored using the *zcIs14 [myo-3p::GFP(mit)]* reporter. To assess mitochondrial content, 30 nematodes per group were immobilised on unseeded NGM plates and imaged at 10× magnification. For mitochondrial morphology, nematodes were mounted on 4% agarose pads, a total of 130 to 150 images of mitochondria within the body wall muscle cells between the pharynx and vulva were acquired at 60× magnification and subsequently categorised into five distinct categories: Class 1, highly abundant mitochondria forming a well-preserved tubular network; Class 2, highly abundant mitochondria with gaps in the network and minor vesiculation; Class 3, relatively sparse mitochondria with network gaps and increased vesiculation; Class 4, sparse and disorganised mitochondria with minor vesiculation; and Class 5, extremely sparse and disorganised mitochondria [70].

Mitophagy was evaluated utilizing the *unc-119(ed3); Ex[myo-3p::tomm-20::Rosella; unc-119(+)]* strain. Images for mitophagy assessment were analysed using the head region of each worm, selecting a muscle-specific area just anterior to the intestine to avoid gut autofluorescence. For each group, 45 nematodes were examined, and both green and red fluorescence signals within the muscle-specific region between the pharynx and the anterior intestine were captured at 60× magnification. The ratio of green to red fluorescence intensity for each individual nematode was then quantified using ImageJ software [28].

The mitochondrial and endoplasmic reticulum (ER) unfolded protein responses (UPR^mt^ and UPR^ER^) were monitored using the *zcIs13[hsp-6p::GFP + lin-15(+)] V* and *zcIs4[hsp-4p::GFP] V* reporter strains, respectively. As previously described [71], a mild mitochondrial and ER stresses were induced prior to the intermittent fasting regimen. Briefly, L4-stage *hsp-4p::GFP* animals were transferred to OP50-seeded plates supplemented with 25 ng/μL tunicamycin (TM) and cultured for 16 h, whereas L4-stage *hsp-6p::GFP* animals were treated on OP50-seeded plates containing 50 μM carbonyl cyanide m-chlorophenyl hydrazone (CCCP) for 8 h. Following the intermittent fasting protocol, the nematodes were transferred and immobilised onto unseeded NGM plates, and images were acquired at 10× magnification.

To monitor SKN-1 activation, the SKN-1 read-out reporter strain *dvIs19[(pAF15)gst-4p::GFP::NLS] III* was employed. After undergoing the respective intermittent fasting regimens, nematodes were immobilised with 40 mM levamisole on unseeded NGM plates and imaged at 10× magnification. The whole-body GFP fluorescence intensity of each nematode was measured using ImageJ software [71].

The nuclear localisation of DAF-16 was evaluated using the strain *zIs356[daf-16p::daf-16a/b::GFP + rol-6(su1006)]*. For each group, 45 nematodes were mounted on 4% agarose pads as described above and imaged at 10× magnification. The captured images were subsequently classified into three distinct categories: nuclear, characterised by robust *daf-16p*::GFP accumulation within the nuclei; intermediate, characterised by incomplete nuclear accumulation with visible punctate fluorescence in the cytoplasm; and cytosolic, characterised by a diffuse *daf-16p*::GFP distribution throughout the cytoplasm [8].

### 4.10 Statistical analysis

Statistical data were presented as mean ± SEM. Statistical differences between two independent groups were assessed using a two-tailed unpaired Student's t-test after confirmation of normal distribution with GraphPad Prism. One-way or two-way analysis of variance (ANOVA) was applied as appropriate when more than two groups were analysed. Survival and lifespan data were evaluated by Kaplan–Meier survival curves followed by a log-rank test, and the mean ± SEM of each condition are detailed in Tables S2–4. Pairwise comparisons were performed using log-rank tests with Holm-adjusted correction for multiple testing. The chi-square test was employed to assess differences in categorical distributions. All statistical analyses were conducted using GraphPad Prism version 10.5. A p-value less than 0.05 was considered statistically significant. Significance levels were defined as follows: ∗p ≤ 0.05, ∗∗p ≤ 0.01, ∗∗∗p ≤ 0.001, and ∗∗∗∗p ≤ 0.0001.

## CRediT authorship contribution statement

**Penglin Li:** Conceptualization, Data curation, Formal analysis, Investigation, Methodology, Visualization, Writing – original draft, Writing – review & editing. **Yating Zheng:** Formal analysis, Methodology, Writing – review & editing. **Jose C. Casas-Martinez:** Formal analysis, Methodology, Writing – review & editing. **Qin Xia:** Formal analysis, Methodology, Writing – review & editing. **Antonio Miranda-Vizuete:** Methodology, Resources, Writing – review & editing. **Katarzyna Goljanek-Whysall:** Formal analysis, Methodology, Resources, Supervision, Writing – review & editing. **Brian McDonagh:** Conceptualization, Formal analysis, Investigation, Methodology, Resources, Supervision, Writing – original draft, Writing – review & editing.

## Declaration of competing interest

The authors declare that they have no known competing financial interests or personal relationships that could have appeared to influence the work reported in this paper.

## Data Availability

Data will be made available on request.
