# Supplementary material for: Fasting requires Peroxiredoxin 2 and Peroxiredoxin 6 to coordinate redox dependent mitochondrial and lipid remodelling in Caenorhabditis elegans
Source: Redox Biol. 2026 May 22;95:104231. doi: 10.1016/j.redox.2026.104231 (PMC13276316; doi:10.1016/j.redox.2026.104231)
Supplement: Multimedia component 1 [file mmc1.docx]

**Supplemental information:**


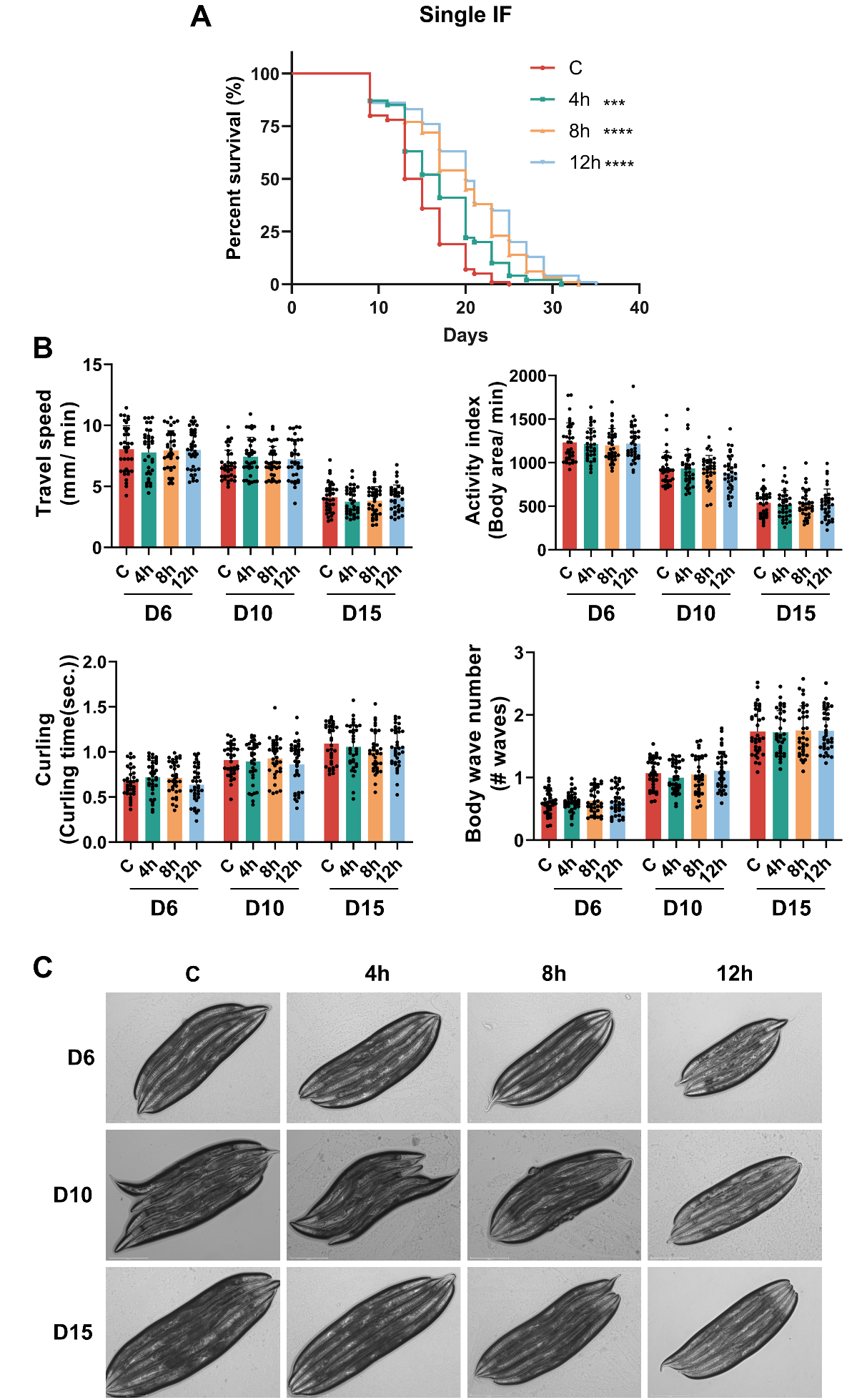


**Fig. S1. A single fasting intervention increases lifespan but does not improve physical activity during aging.** **(A)** Lifespan of the wild type N2 following a single fasting intervention for 4 h, 8 h, or 12 h on adult day 1. ****p ≤ 0.0001 (n = 105). **(B)** Assessment of physiological activity (Travel speed, Activity index) and frailty (Curling time, Body wave number) using the CeleST system at days 6, 10, and 15 following the single fasting intervention. At least 30 animals for each condition were measured. **(C)** Representative images assessing morphological parameters including body width, body length, and body area of wild type N2 nematodes at days 6, 10, and 15 following the 5-day IF (n = 30).


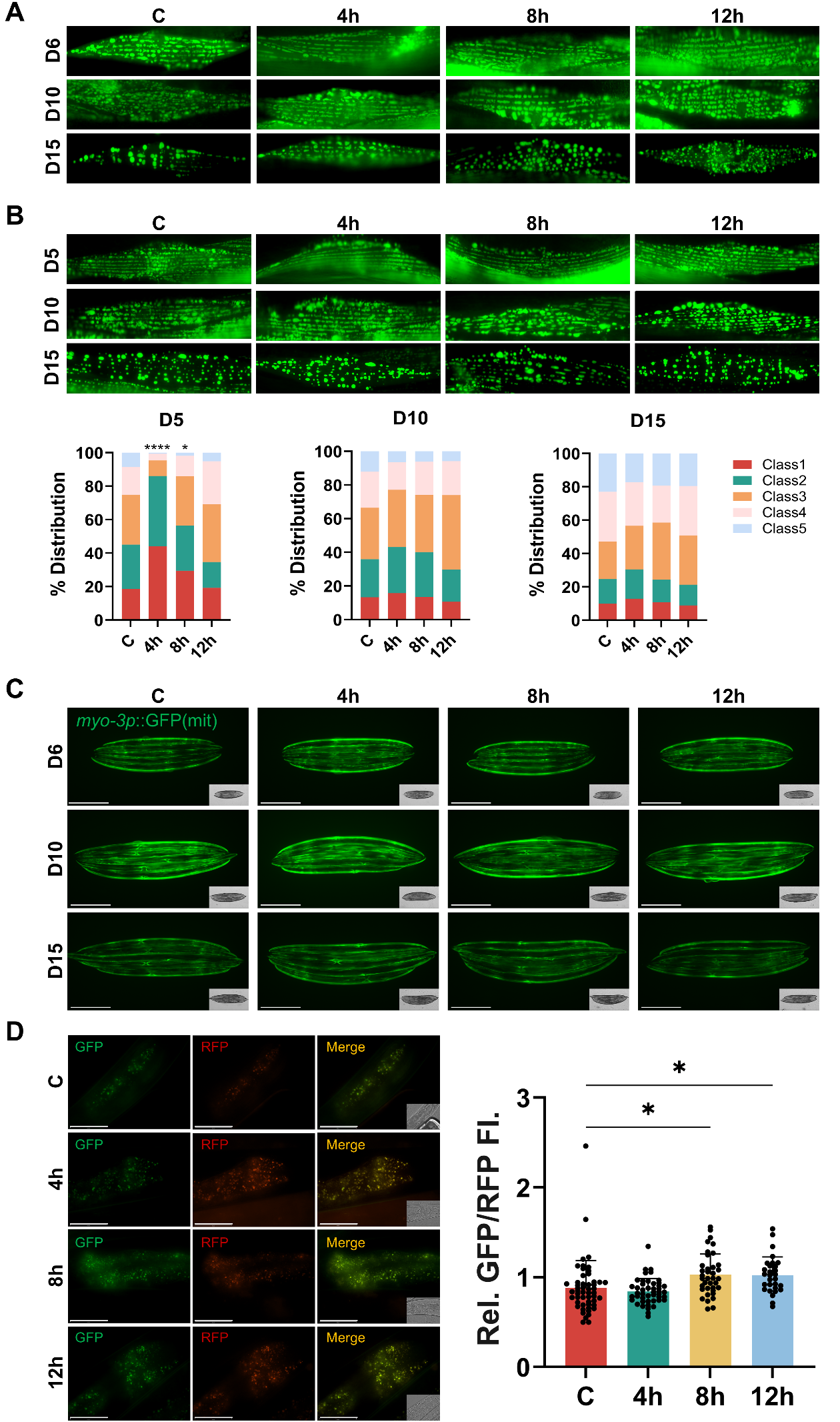


**Fig. S2: A single fasting intervention transiently alters mitochondrial morphology and decreases mitophagy.** **(A)** Representative images of mitochondrial morphology within body wall muscle at days 6, 10, and 15 following the 5-day IF protocol. **(B)** Representative images and quantification of mitochondrial morphology in body wall muscle at days 5, 10, and 15 following a single fasting intervention on adult day 1. *p ≤ 0.05 and ****p ≤ 0.0001(Data represent the percentage distribution of 130-150 images per group). **(C)** Representative images assessing muscle mitochondrial content of the *zcIs14[myo-3p::GFP(mit)]* reporter at days 6, 10, and 15 following the 5-day IF protocol. (Scale bars: 275 μm). **(D)** Representative images of day 2 adult *unc-119(ed3)*; *Ex[myo-3p::tomm-20::Rosella; unc-119(+)]* mitophagy reporter following a single fasting intervention on day 1 adult (Scale bars: 50 μm). *p ≤ 0.05 (n = 30-45).


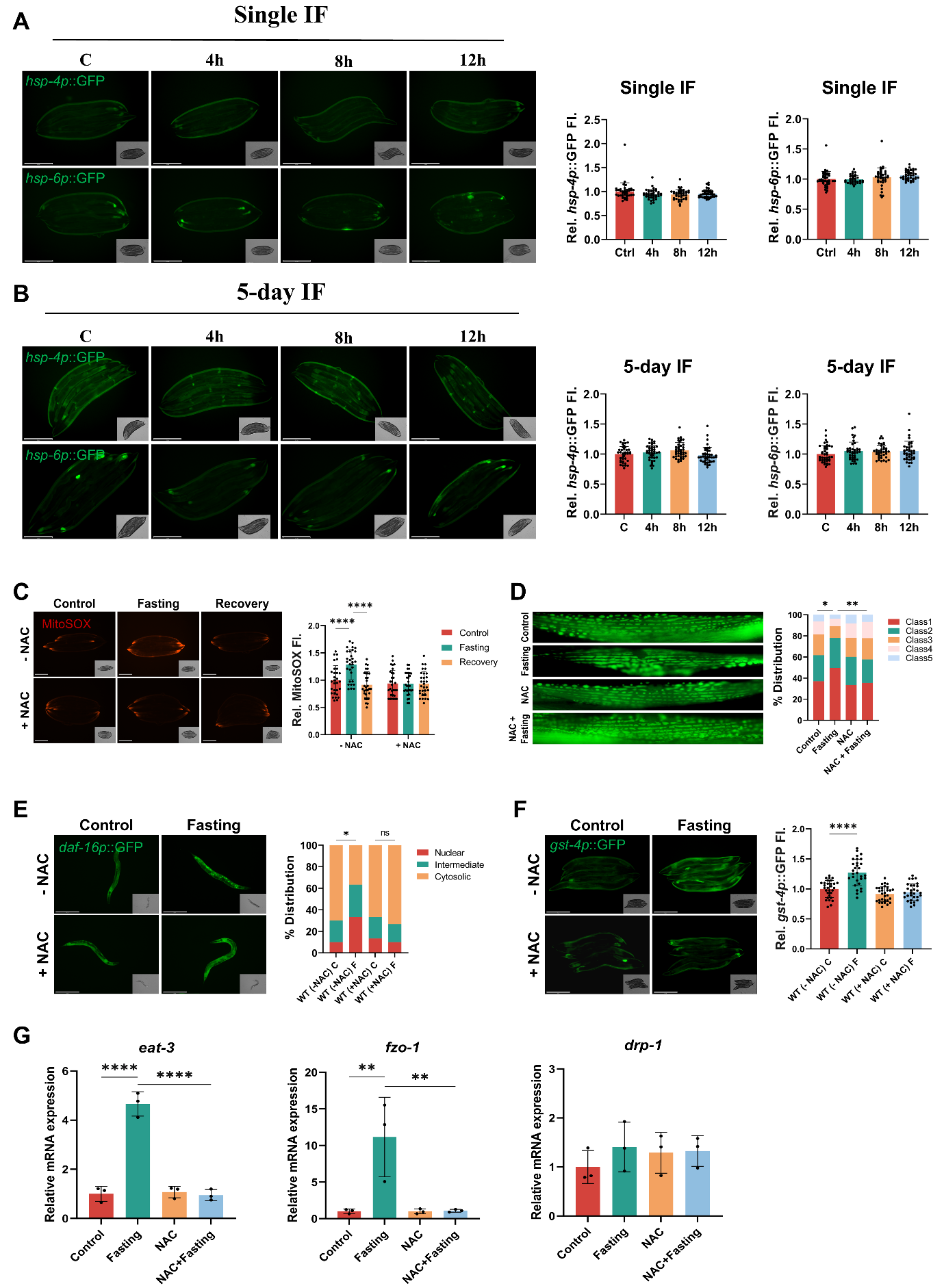


**Fig. S3: Fasting interventions do not activate the endoplasmic reticulum or mitochondrial unfolded protein responses.** **Moreover**, **fasting induced transient ROS promotes mitochondrial fusion. (A)** Activation of UPR^ER^ and UPR^mt^ assessed by *zcIs4[hsp-4p::GFP] V and zcIs13[hsp-6p::GFP + lin-15(+)] V* reporter on adult day 2 following a single fasting intervention (n = 40-45), (Scale bars: 275 μm). **(B)** UPR^ER^ and UPR^mt^ activation on adult day 6 following a 5-day IF protocol using the *zcIs4[hsp-4p::GFP] V and zcIs13[hsp-6p::GFP + lin-15(+)] V* reporter (n = 40-45), (Scale bars: 275 μm). **(C)** Representative images of mitochondrial ROS in N2 wild type strain treated with or without 5 mM of NAC across a fasting and 16 hr-refeeding cycle on adult day 1(Scale bars: 275 μm). ****p ≤ 0.0001(n = 30). **(D)** Representative images of mitochondrial morphology within body wall muscle of *zcIs14[myo-3p::GFP(mit)]* strain treated with or without 5 mM of NAC under control and 4 hr 5-day IF conditions. Images were acquired from day 6 adult worms. Data represent the percentage distribution of 130-150 images per group. *p ≤ 0.05 and **p ≤ 0.01. **(E, F)** DAF-16 nuclear localization and SKN-1 activation in adult day 6 nematodes treated with or without 5 mM of NAC following the 4 hr 5-day IF. (n = 30), (Scale bars: 275 μm). **(G)** Relative mRNA expression levels of the mitochondrial dynamics regulators *eat-3*, *fzo-1*, and *drp-1* in day 6 adult wild type treated with or without 5 mM of NAC under control and 4 hr 5-day IF conditions. Data represent the mean ± SEM (n = 3). **p ≤ 0.01 and ****p ≤ 0.0001.


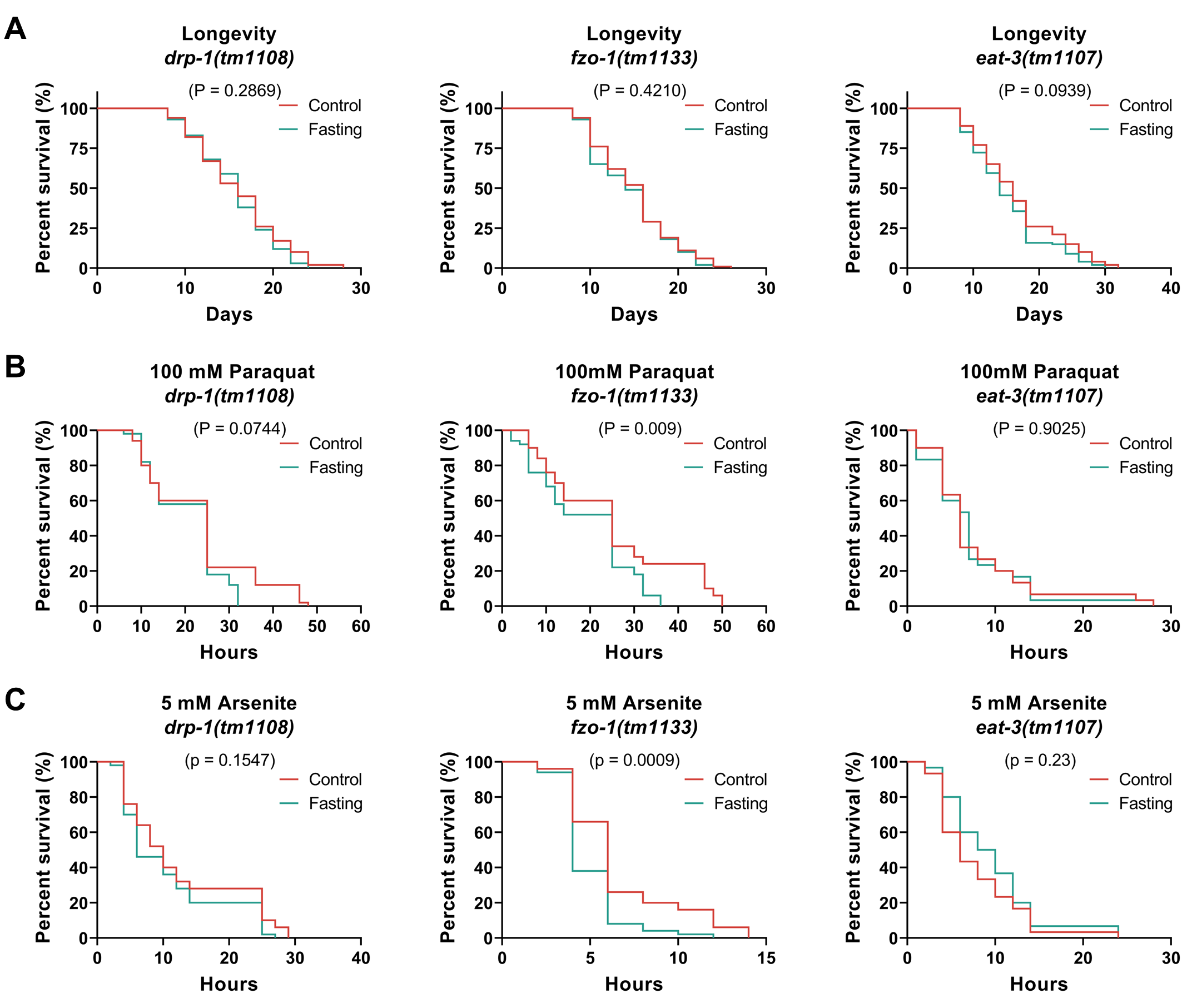


**Fig. S4: Mitochondrial fission and fusion genes are required for fasting-mediated longevity and stress resistance.** Survival assays of *drp-1 (tm1108)*, *fzo-1 (tm1133)*, and *eat-3 (tm1107)* mutant strains subjected to a 4 hr 5-day IF protocol compared to controls. **(A)** Lifespan assays (Longevity). Survival assays performed on adult day 6 under oxidative stress induced by 100 mM paraquat **(B)** or 5 mM arsenite **(C)**. For all panels, Kaplan–Meier survival plots represent experiments initiated with 50 animals per group. P-values are indicated on the graphs, determined by the Log-rank (Mantel-Cox) test.


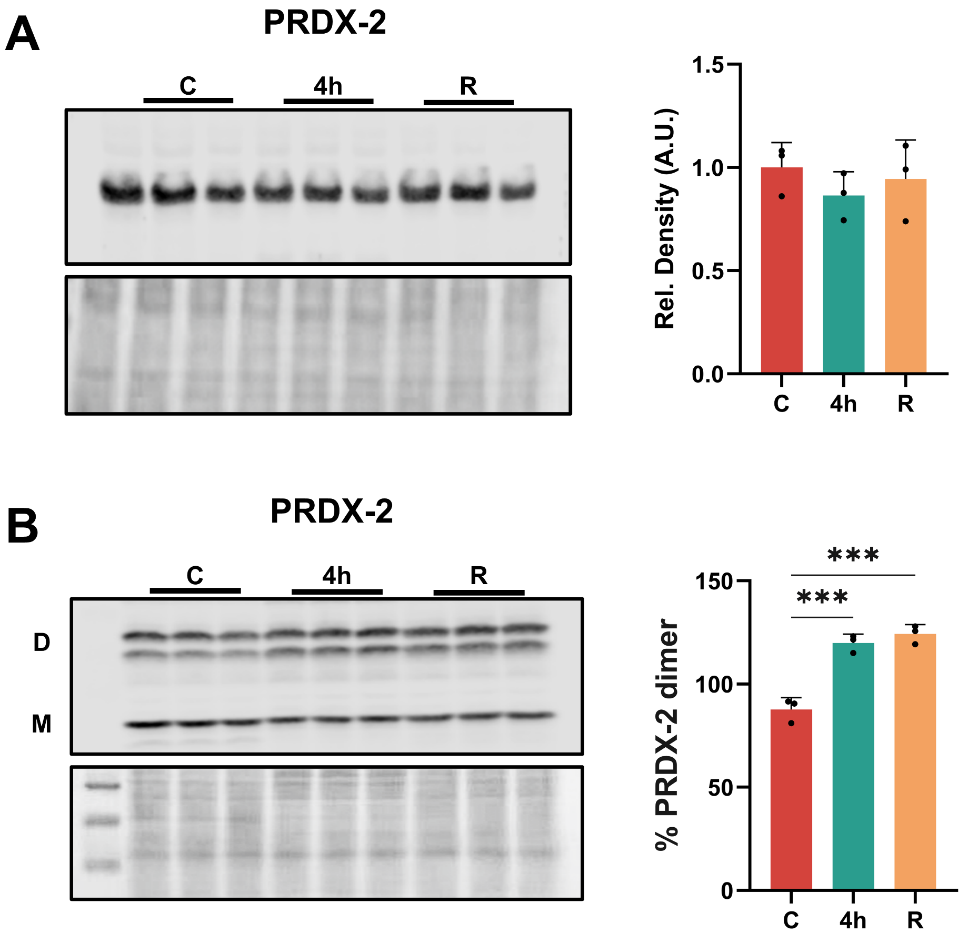


**Fig. S5: A 4 hr fasting intervention alters the redox state of PRDX-2 without changing overall protein abundance in N2 wild type strain.** **(A)** Overall PRDX-2 protein levels in day 1 adult worms under control, 4 hr fasting, and recovery (16 hr) conditions. **(B)** The PRDX-2 redox state in day 1 adults assessed by non-reducing gel electrophoresis under control, 4 hr fasting, and 16 hr-recovery conditions. ***p ≤ 0.001.


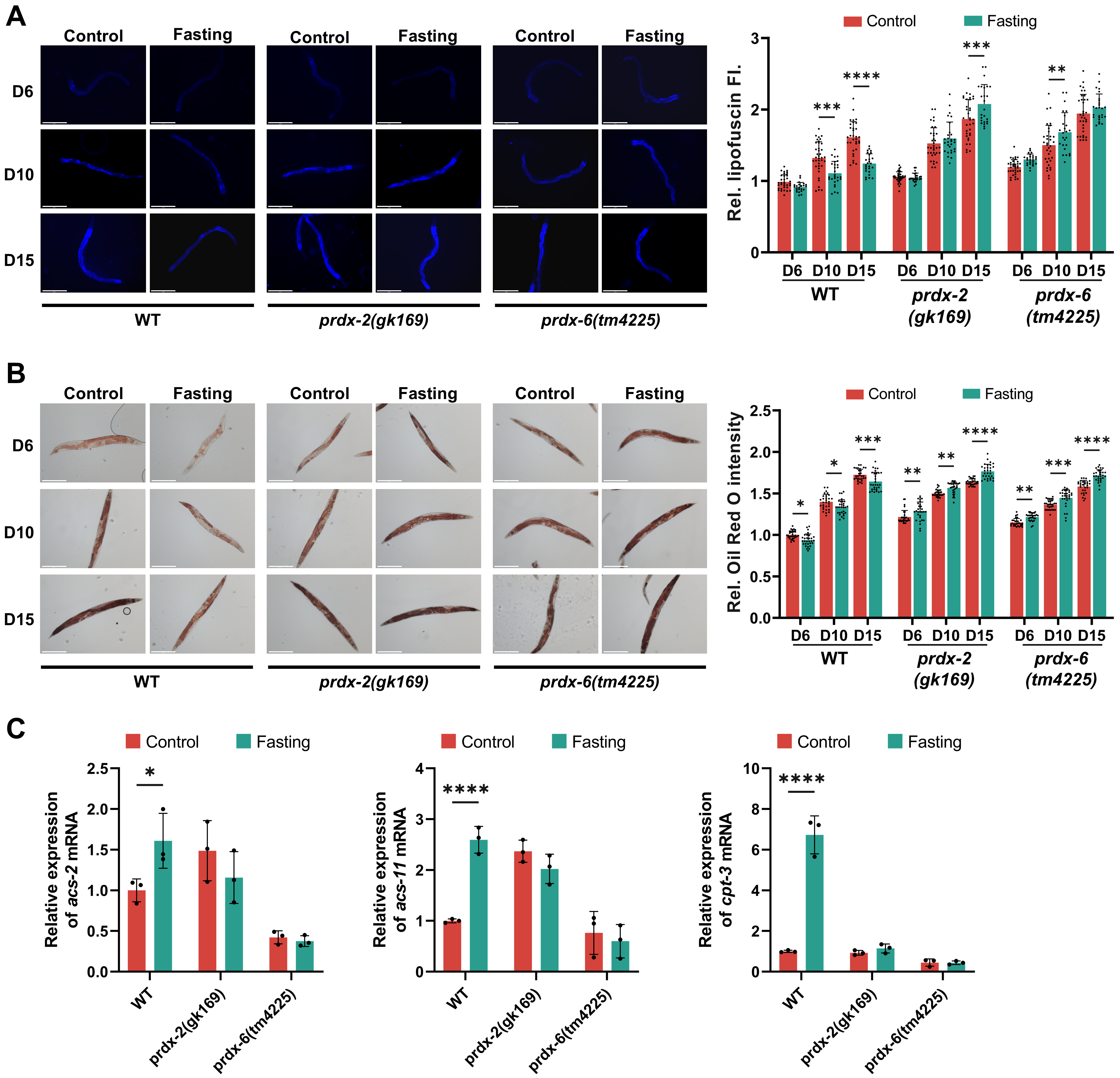


**Fig. S6: Fasting reduces age-related lipofuscin and lipid accumulation in the N2 wild-type strain but exacerbates accumulation in *prdx-2 (gk169)* and *prdx-6* (*tm4225)*** **mutants.** **(A)** Representative images of age-related lipofuscin accumulation in wild-type, *prdx-2*, and *prdx-6* mutant strains at days 6, 10, and 15 following a 4 hr 5-day IF. Scale bars, 275 μm. **p ≤ 0.01, ***p ≤ 0.001, and ****p ≤ 0.0001 (n = 28-34). **(B)** Representative images of Oil Red O staining in wild type, *prdx-2(gk169)*, and *prdx-6(tm4225)* strains at days 6, 10, and 15 following the 4 hr 5-day fasting protocol. Scale bars, 275 μm. *p ≤ 0.05, **p ≤ 0.01, ***p ≤ 0.001, and ****p ≤ 0.0001(n = 28-34). **(C)** Relative mRNA expression levels of the fatty acid β-oxidation related genes *acs-2*, *acs-11*, and *cpt-3* in day 6 adult wild type, *prdx-2* and *prdx-6* mutants following the 4 hr 5-day IF. Data represent the mean ± SEM (n = 3). *p ≤ 0.05, and ****p ≤ 0.0001.


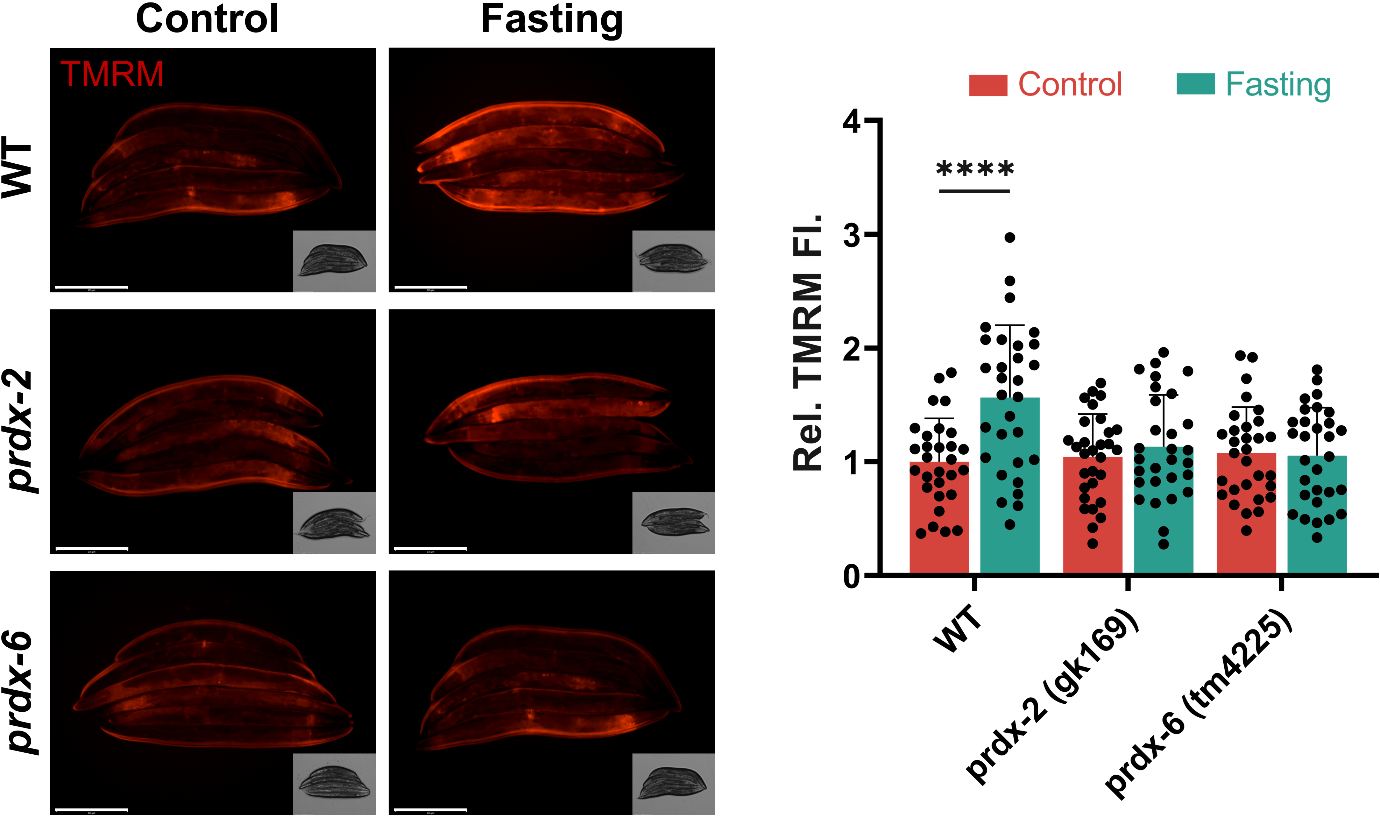


**Fig. S7: PRDX-2 and PRDX-6 are required for the promoted mitochondrial membrane potential following the 4 hr 5-day fasting**. Representative images and quantification of mitochondrial membrane potential assessed by TMRM staining in wild type N2, *prdx-2(gk169)*, and *prdx-6(tm4225)* strains on day 6 following 4 hr 5-day intermittent fasting. Scale bars, 275 μm. ****p ≤ 0.0001(n = 30).


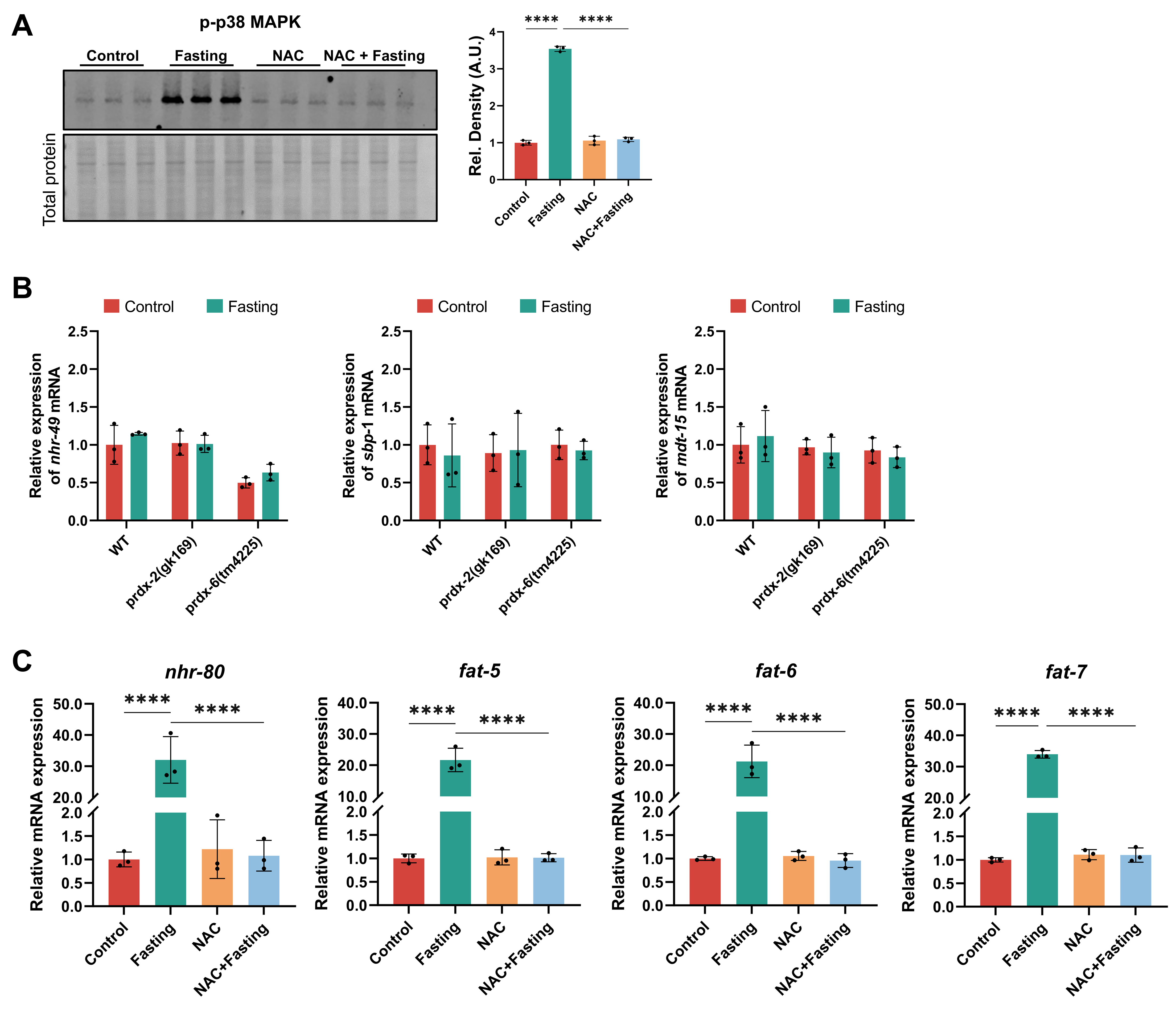


**Fig. S8: Fasting induced ROS activates the p38 MAPK and NHR-80 signaling pathways.** **(A)** Western blot images and quantification of phosphorylated p38 MAPK in day 6 adult wild type treated with or without 5 mM of NAC following the 4 hr 5-day IF. **(B)** Relative mRNA expression levels of the lipid metabolism regulators *nhr-49*, *sbp-1*, and *mdt-15* in day 6 adult wild type, *prdx-2(gk169)*, and *prdx-6(tm4225)* mutant strains under control and 4 hr 5-day IF conditions. Data represent the mean ± SEM (n = 3). **(C)** Relative mRNA expression levels of lipid desaturation regulators *nhr-80*, *fat-5*, *fat-6,* and *fat-7* in day 6 adult wild type treated with or without 5 mM of NAC following the 4 hr 5-day IF. Data represent the mean ± SEM (n = 3).


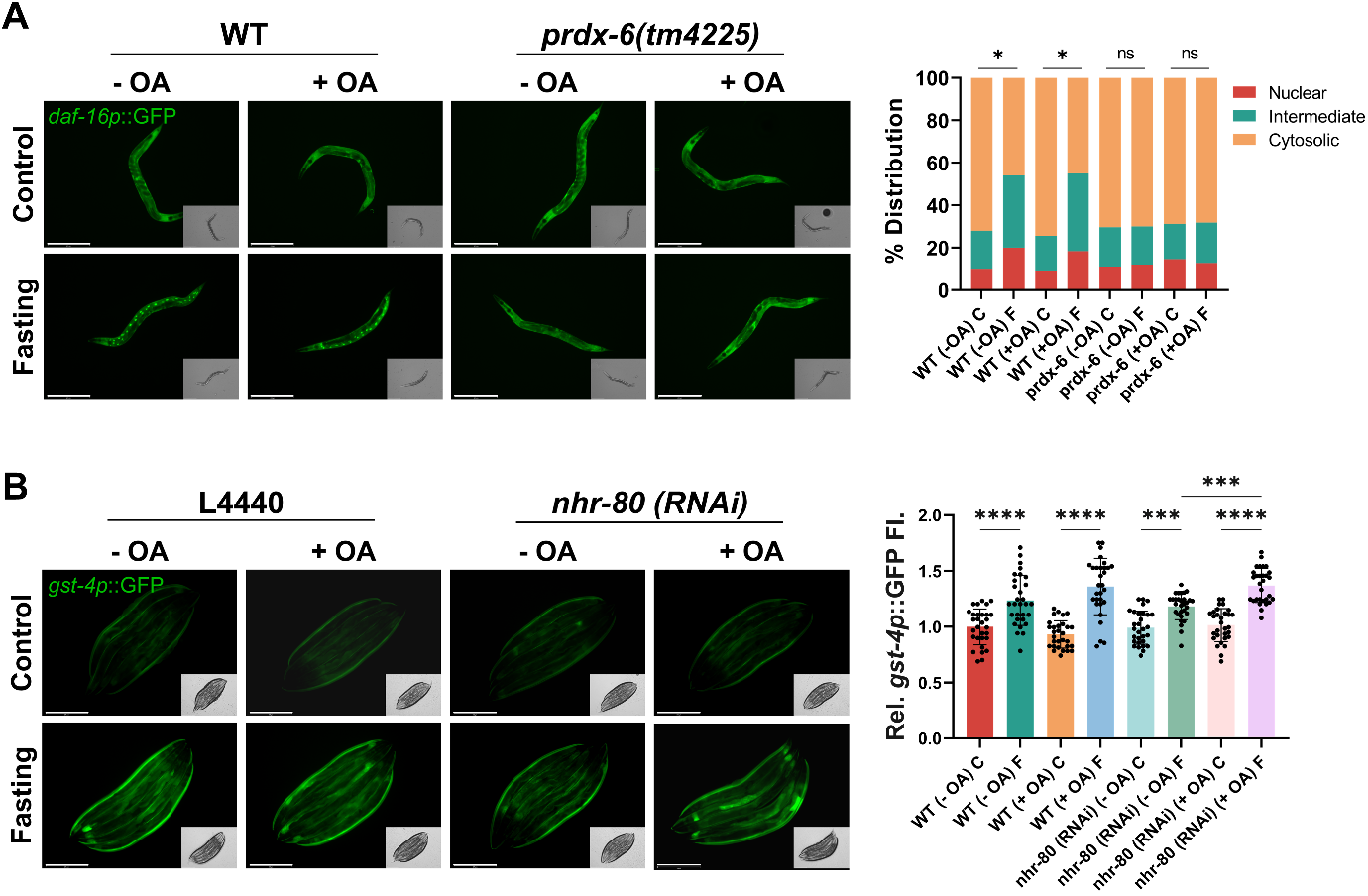


**Fig. S9: A) OA supplementation did not rescue nuclear localisation of DAF-16 in *prdx-6* (*tm4225)* mutant strain following 4 hr 5-day IF.** Representative images of DAF-16 nuclear localisation reporter *zIs356[daf-16p::daf-16a/b::GFP + rol-6(su1006)]* under the wild type and *prdx-6(tm4225)* mutant backgrounds treated with or without 0.8 mM of OA following the 4 hr 5-day IF (Scale bars: 275 μm). Images were acquired from day 6 adults and categorized as: cytosolic, intermediate, and nuclear localization. Data represent the percentage distribution of 45-50 animals per group. *p ≤ 0.05. **(B) OA supplementation can rescue SKN-1 activation as a result of fasting following RNAi *nhr-80* knockdown.** Representative images of SKN-1 activation reporter *dvIs19[(pAF15)gst-4p::GFP::NLS] III* acquired in control and *nhr-80 (RNAi)* treated with or without 0.8 mM of OA following the 4 hr 5-day IF (Scale bars: 275 μm). ***p ≤ 0.001, and ****p ≤ 0.0001 (n = 30).


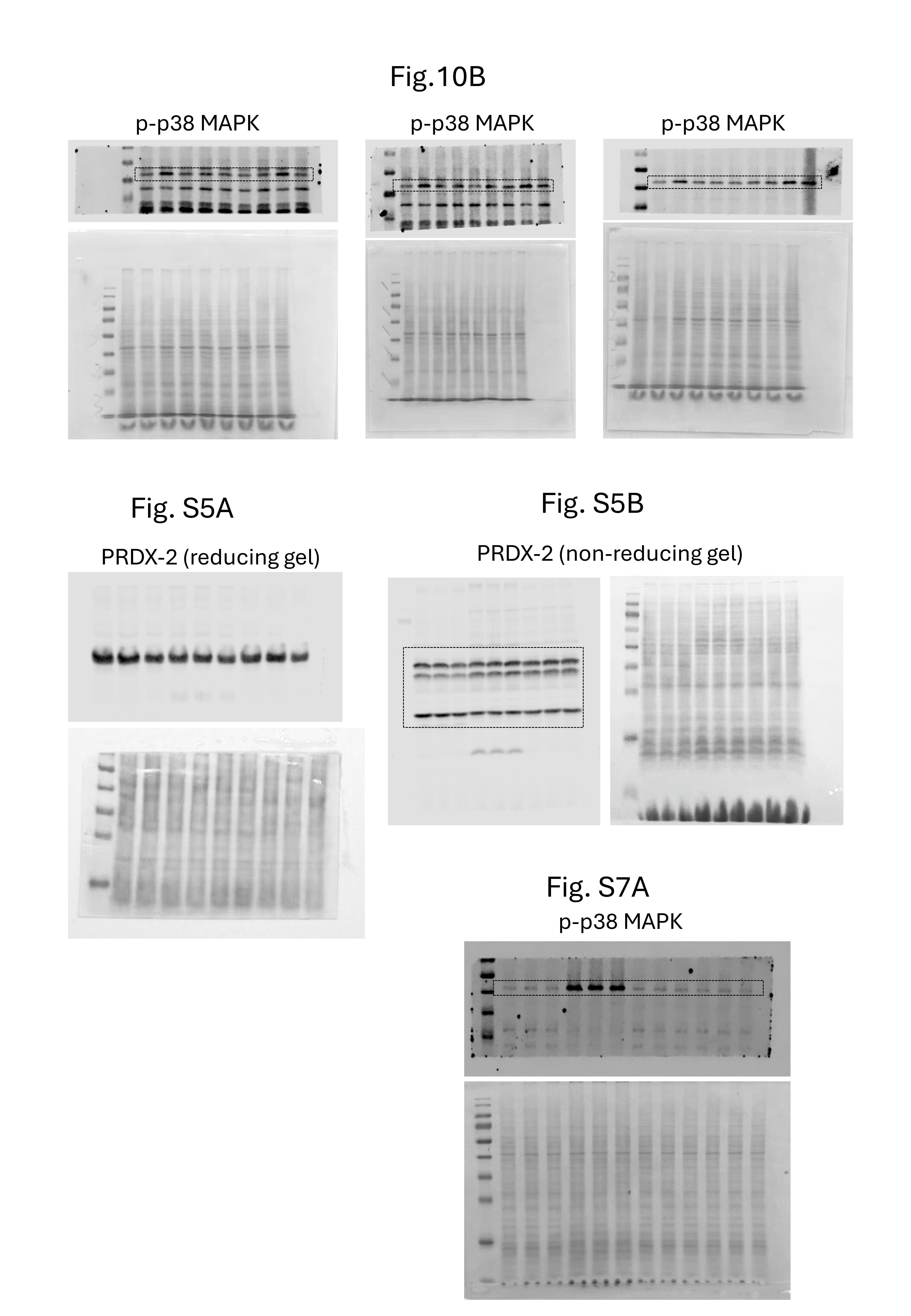


**Fig. S10: Uncropped western blot images corresponding to Fig. 10B, S5A, S5B, and S8A.**


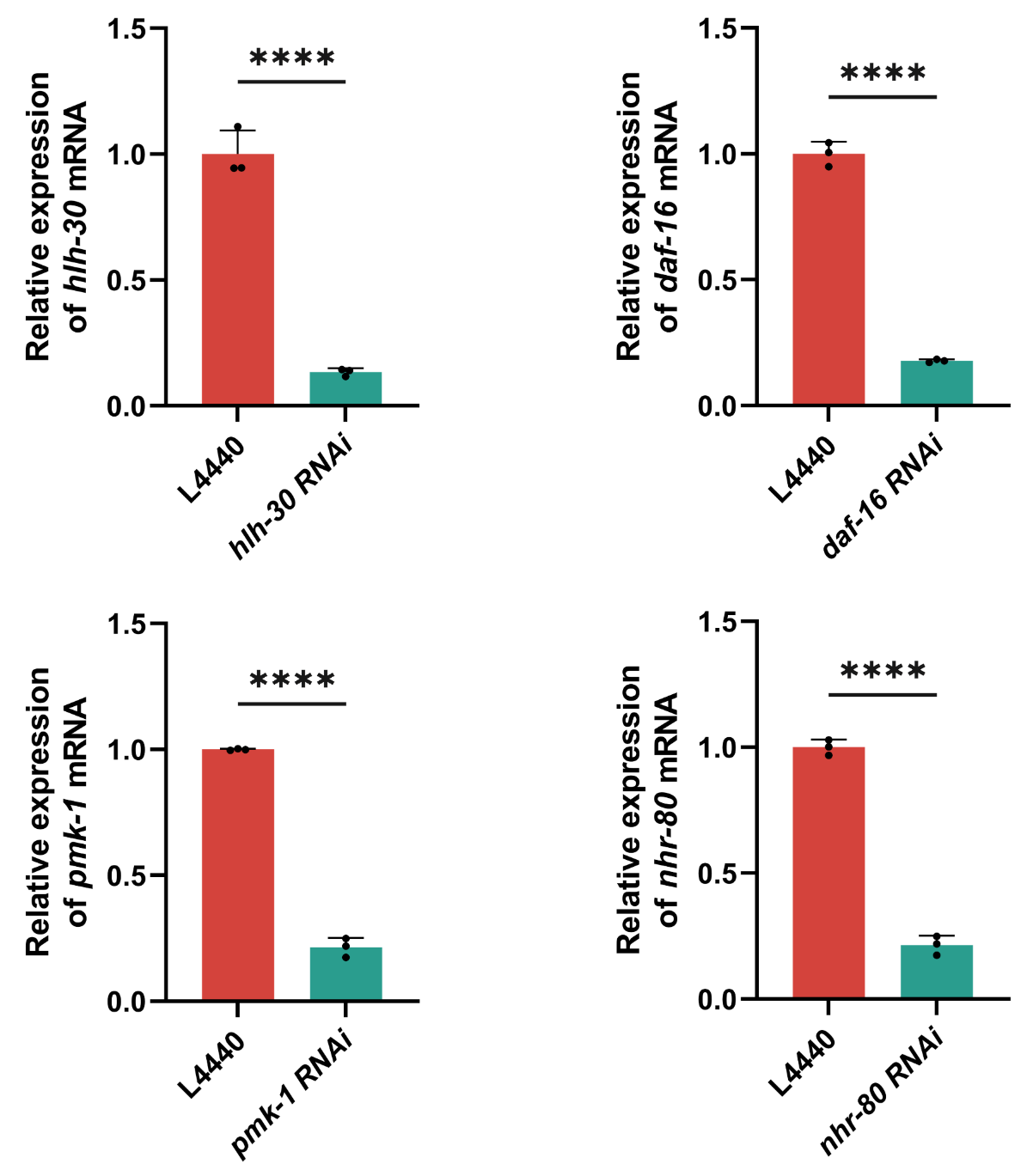


**Fig. S11: Validation of RNA interference (RNAi) knockdown efficiency for target genes.** Relative mRNA expression levels of *hlh-30*, *daf-16*, *pmk-1*, and *nhr-80* analysed by qPCR. Wild-type nematodes were fed *E. coli* HT115 expressing target-specific double-stranded RNA (dsRNA) or the L4440 empty vector control. Data represent the mean ± SEM (n = 3). ****p ≤ 0.0001 compared to the L4440 control.

**Table S1.** Primers for genotyping and qPCR

| **Gene** | **Primer** |
| --- | --- |
| **Genotyping** | |
| *prdx-2* | OF 5’-CGCTCCTCCTAAACGTTGTAGTC-3’ |
|  | OR 5’-CTAGGGATACGGGGGAAATTAG-3’ |
|  | IR 5’-GGATATTGAAACCCAACGGCAACG-3’ |
| *prdx-6* | OF 5’-GATGGCTGATGCTGTTCAG-3’ |
|  | IF 5’-CGGAGGGAATCTGTCTTTC-3’ |
|  | OR 5’-GCGCTCCGTAAATCTACAC-3’ |
| **qPCR** | |
| *cdc-42* | Fw 5’-AGCCATTCTGGCCGCTCTCG-3’ |
|  | Rv 5’-GCAACCGCTTCTCGTTTGGC-3’ |
| *drp-1* | Fw 5’-ACAAAACTGACCCTGTGGGG-3’ |
|  | Rv 5’-TAGGCTCCGAAGTAGCGAAA-3’ |
| *fzo-1* | Fw 5’-GCTTTCTGCAGGTTGAAGGT-3’ |
|  | Rv 5’-CGACACCAGGGCTATCAAGT-3’ |
| *eat-3* | Fw 5’-AGAGCATCGAAACCGGATGG-3’ |
|  | Rv 5’-GCGTCAGCATAGCTTCTTCG-3’ |
| *prdx-2* | Fw 5’-CATTCCAGTTCTCGCTGAC-3’ |
|  | Rv 5’-ATGATGAAGAGTCCACGGA-3’ |
| *prdx-3* | Fw 5’-GTTCCGTTCTCTTGGAGCTG-3’ |
|  | Rv 5’-CTTGTTGAAATCAGCGAGCA-3’ |
| *prdx-6* | Fw 5’-GGAGAACAATGGCTGATGC-3’ |
|  | Rv 5’-ATCTGAACATGGCGTTTGC-3’ |
| *daf-16* | Fw 5’-AAAGAGCTCGTGGTGGGTTA-3’ |
|  | Rv 5’-TTCGAGTTGAGCTTTGTAGTCG-3’ |
| *hlh-30* | Fw 5’-CTCATCGGCCGGCGCTCATC-3’ |
|  | Rv 5’-AGAACGCGATGCGTGGTGGG-3’ |
| *spg-7* | Fw 5’-CCGTTGTCGTTTGAGACACC-3’ |
|  | Rv 5’-CGGCGAAGTGCGTTCATTAC-3’ |
| *ppgn-1* | Fw 5’-ATGCTTCTACACCGCTCCAC-3’ |
|  | Rv 5’-GTGGAAATCTGCGAGCACT-3’ |
| *hnr-49* | Fw 5’-TCCGAGTTCATTCTCGACG-3’ |
|  | Rv 5’-GGATGAATTGCCAATGGAGC-3’ |
| *nhr-80* | Fw 5’-ATCACCGACGAGATCATGCC-3’ |
|  | Rv 5’-TCGAAACCCCCTTGAAAGCA-3’ |
| *sbp-1* | Fw 5’-AATCTGGGTTTGGCGGTTGGC-3’ |
|  | Rv 5’-CGAGCGACTTCTTTGTGTGAATGC-3’ |
| *mdt-15* | Fw 5’-GGAAATCCGTACAATCAGCAG-3’ |
|  | Rv 5’-CAAGAACTGATGAAGGACCG-3’ |
| *fat-5* | Fw 5’-CGATTTGTACGAGGATCCGGTG-3’ |
|  | Rv 5’-CAGTGGGAGACACTGTTGATGC-3’ |
| *fat-6* | Fw 5’-TCAACAGCGCTGCTCACTAT-3’ |
|  | Rv 5’-TTCGACTGGGGTAATTGAGG-3’ |
| *fat-7* | Fw 5’-CAACAGCGCTGCTCACTATT-3’ |
|  | Rv 5’-CACCAACGGCTACAACTGTG-3’ |
| *pmk-1* | Fw 5’-ATGGAACTGTTTGTGCTGCTG-3’ |
|  | Rv 5’-TCACGATATGTACGACGGGC-3’ |
| *acs-2* | Fw 5’-GCAGCCTCGCTCTACACTCT-3’ |
|  | Rv 5’-GACTCCTGCAAATGCACATG-3’ |
| *acs-11* | Fw 5’-TCCGATCCTTCGAAGCTCCT-3’ |
|  | Rv 5’-GAGCAGCTGTATCGGTCGTT-3’ |
| *cpt-3* | Fw 5’-GTTGGCGAAAATCGTCTCCG-3’ |
|  | Rv 5’-AGGAAGAATTTCCGGCGGTT-3’ |

**Table S2** Data from lifespan assays

| Figure | Group  (Strain and condition) | # Deaths/ # Total | Mean lifespan | Log-rank test, *p*-value (Compared to control) |
| --- | --- | --- | --- | --- |
| 1B | Wild type (Ctrl) | 102/105 | 16.06 ± 0.63 |  |
|  | Wild type (4h) | 100/105 | 20.38 ± 0.75 | < 0.0001 |
|  | Wild type (8h) | 103/105 | 20.10 ± 0.73 | < 0.0001 |
|  | Wild type (12h) | 99/105 | 21.57 ± 0.71 | < 0.0001 |
| 5A | Wild type Control | 105/105 | 15.74 ± 0.54 |  |
|  | Wild type Fasting | 103/105 | 19.20 ± 0.59 | 0.0001 |
|  | *prdx-2* Control | 102/105 | 12.60 ± 0.28 |  |
|  | *prdx-2* Fasting | 100/105 | 12.64 ± 0.29 | 0.8529 |
|  | *prdx-3* Control | 101/105 | 14.62 ± 0.47 |  |
|  | *prdx-3* Fasting | 98/105 | 15.98 ± 0.54 | 0.0397 |
|  | *prdx-6* Control | 99/105 | 15.28 ± 0.38 |  |
|  | *prdx-6* Fasting | 98/105 | 13.82 ± 0.30 | 0.0018 |
| 9H | Wild type (L4440) C | 105/105 | 16.41 ± 0.41 |  |
|  | Wild type (L4440) F | 101/105 | 19.34 ± 0.48 | < 0.0001 |
|  | *daf-16* (RNAi) C | 105/105 | 15.96 ± 0.38 |  |
|  | *daf-16* (RNAi) F | 100/105 | 13.33 ± 0.33 | < 0.0001 |
|  | *hlh-30* (RNAi) C | 101/105 | 16.24 ± 0.38 |  |
|  | *hlh-30* (RNAi) F | 98/105 | 12.96 ± 0.33 | < 0.0001 |
| 10A | Wild type C | 99/105 | 15.50 ± 0.50 |  |
|  | Wild type F | 102/105 | 18.02 ± 0.55 | 0.0017 |
|  | *prdx-2* C | 98/105 | 12.46 ± 0.34 |  |
|  | *prdx-2* F | 99/105 | 12.22 ± 0.32 | 0.5631 |
|  | *prdx-6* C | 105/105 | 14.98 ± 0.45 |  |
|  | *prdx-6* F | 105/105 | 13.82 ± 0.40 | 0.0182 |
|  | *prdx-2; prdx-6* C | 103/105 | 12.20 ± 0.34 |  |
|  | *prdx-2; prdx-6* F | 101/105 | 10.42 ± 0.20 | < 0.0001 |
| 10C | Wild type (L4440) C | 105/105 | 15.62 ± 0.47 |  |
|  | Wild type (L4440) F | 100/105 | 17.10 ± 0.53 | 0.0099 |
|  | Wild type; *pmk-1*(RNAi) C | 99/105 | 13.54 ± 0.32 |  |
|  | Wild type; *pmk-1*(RNAi) F | 103/105 | 13.46 ± 0.32 | 0.8296 |
|  | *prdx-2* (L4440) C | 102/105 | 13.46 ± 0.34 |  |
|  | *prdx-2* (L4440) F | 101/105 | 13.17 ± 0.32 | 0.3077 |
|  | *prdx-2; pmk-1*(RNAi) C | 99/105 | 13.03 ± 0.30 |  |
|  | *prdx-2; pmk-1*(RNAi) F | 103/105 | 13.08 ± 0.30 | 0.8994 |
| 10I | Wild type (L4440) C | 99/105 | 15.62 ± 0.47 |  |
|  | Wild type (L4440) F | 101/105 | 17.10 ± 0.53 | 0.0099 |
|  | Wild type; *nhr-80* (RNAi) C | 105/105 | 15.43 ± 0.47 |  |
|  | Wild type; *nhr-80* (RNAi) F | 100/105 | 13.60 ± 0.36 | 0.0002 |
|  | *prdx-6* (L4440) C | 98/105 | 14.90 ± 0.46 |  |
|  | prdx-6 (L4440) F | 99/105 | 13.72 ± 0.39 | 0.0154 |
|  | *prdx-6; nhr-80* (RNAi) C | 101/105 | 15.46 ± 0.44 |  |
|  | *prdx-6 ;nhr-80* (RNAi) F | 101/105 | 13.77 ± 0.38 | 0.0022 |
| 10J | *prdx-6* C | 98/105 | 14.71 ± 0.44 |  |
|  | *prdx-6* F | 102/105 | 13.60 ± 0.39 | 0.0309 |
|  | *prdx-6* + OA C | 96/105 | 14.48 ± 0.41 |  |
|  | *prdx-6* + OA F | 104/105 | 16.77 ± 0.58 | 0.0001 |
| S1A | Wild type (Ctrl) | 100/105 | 14.64 ± 0.41 |  |
|  | Wild type (4h) | 98/105 | 16.95 ± 0.53 | 0.0002 |
|  | Wild type (8h) | 97/105 | 18.91 ± 0.65 | < 0.0001 |
|  | Wild type (12h) | 102/105 | 20.30 ± 0.66 | < 0.0001 |
| S4A | *dpr-1* Control | 99/105 | 15.96 ± 0.49 |  |
|  | *dpr1* Fasting | 100/105 | 15.60 ± 0.43 | 0.2869 |
|  | *fzo-1* Control | 102/105 | 15.00 ± 0.44 |  |
|  | *fzo-1* Fasting | 97/105 | 14.48 ± 0.44 | 0.4210 |
|  | *eat-3* Control | 99/105 | 16.62 ± 0.65 |  |
|  | *eat-3* Fasting | 101/105 | 15.24 ± 0.57 | 0.0939 |

Number of worms represents the number of dead worms scored relative to total number of worms initially started with. The difference in number is indicative of censored worms.

**Table S3** Data from Paraquat-induced oxidative stress survival assays

| Figure | Group  (Strain and condition) | n | Mean Survival in Paraquat | Log-rank test, *p*-value (Compared to control) |
| --- | --- | --- | --- | --- |
| 5B | Wild type Control | 50 | 24.02 ± 1.86 |  |
|  | Wild type Fasting | 50 | 31.36 ± 2.3 | 0.0055 |
|  | *prdx-2* Control | 50 | 27.54 ± 1.73 |  |
|  | *prdx-2* Fasting | 50 | 22.22 ± 1.59 | 0.0044 |
|  | *prdx-3* Control | 50 | 24.40 ± 2.19 |  |
|  | *prdx-3* Fasting | 50 | 32.12 ± 2.38 | 0.0021 |
|  | *prdx-6* Control | 50 | 34.32 ± 1.71 |  |
|  | *prdx-6* Fasting | 50 | 21.98 ± 1.78 | < 0.0001 |
| 9H | Wild type (L4440) C | 50 | 23.96 ± 1.84 |  |
|  | Wild type (L4440) F | 50 | 30.30 ± 1.95 | 0.0091 |
|  | *daf-16* (RNAi) C | 50 | 15.46 ± 1.32 |  |
|  | *daf-16* (RNAi) F | 50 | 10.62 ± 1.05 | 0.0009 |
|  | *hlh-30* (RNAi) C | 50 | 15.66 ± 1.42 |  |
|  | *hlh-30* (RNAi) F | 50 | 9.78 ± 1.10 | 0.0003 |
| S4B | *dpr-1* Control | 50 | 23.14 ± 1.68 |  |
|  | *dpr1* Fasting | 50 | 19.46 ± 1.20 | 0.0744 |
|  | *fzo-1* Control | 50 | 24.94 ± 2.09 |  |
|  | *fzo-1* Fasting | 50 | 18.70 ± 1.52 | 0.009 |
|  | *eat-3* Control | 30 | 7.70 ± 1.13 |  |
|  | *eat-3* Fasting | 30 | 7.17 ± 1.01 | 0.9025 |

**Table S4** Data from Sodium Arsenite induced oxidative stress survival assays

| Figure | Group  (Strain and condition) | n | Mean Survival in Sodium Arsenite | Log-rank test, *p*-value (Compared to control) |
| --- | --- | --- | --- | --- |
| 5C | Wild type Control | 50 | 7.64 ± 0.7 |  |
|  | Wild type Fasting | 50 | 12.08 ± 1.31 | 0.0045 |
|  | *prdx-2* Control | 50 | 5.76 ± 0.67 |  |
|  | *prdx-2* Fasting | 49 | 3.12 ± 0.24 | < 0.0001 |
|  | *prdx-3* Control | 50 | 7.16 ± 0.67 |  |
|  | *prdx-3* Fasting | 50 | 10.38 ± 1.03 | 0.0112 |
|  | *prdx-6* Control | 50 | 5.86 ± 0.72 |  |
|  | *prdx-6* Fasting | 50 | 3.64 ± 0.36 | 0.0094 |
| 9H | Wild type (L4440) C | 50 | 13.12 ± 1.11 |  |
|  | Wild type (L4440) F | 50 | 17.52 ± 1.28 | 0.0012 |
|  | *daf-16* (RNAi) C | 50 | 8.00 ± 0.72 |  |
|  | *daf-16* (RNAi) F | 50 | 6.82 ± 0.57 | 0.2245 |
|  | *hlh-30* (RNAi) C | 50 | 8.50 ± 0.78 |  |
|  | *hlh-30* (RNAi) F | 50 | 6.32 ± 0.52 | 0.0085 |
| S4C | *dpr-1* Control | 50 | 12.68 ± 1.25 |  |
|  | *dpr1* Fasting | 50 | 10.72 ± 1.12 | 0.1547 |
|  | *fzo-1* Control | 50 | 6.60 ± 0.44 |  |
|  | *fzo-1* Fasting | 50 | 4.92 ± 0.25 | 0.0009 |
|  | *eat-3* Control | 30 | 7.73 ± 0.88 |  |
|  | *eat-3* Fasting | 30 | 9.53 ± 0.96 | 0.23 |

**Reagents and Resources**

| **Reagent or Resource** | **Source** | **Identifier** |
| --- | --- | --- |
| **Antibodies** | | |
| rabbit anti-Peroxiredoxin 2 | Elizabeth Veal lab [1] | N/A |
| rabbit anti-Phospho-p38 MAPK | Cell Signalling Technology | Cat# 4511 |
| IRDye 800CW Goat anti-Rabbit IgG | LI-COR Biosciences | Cat# 926-32211 |
| **Bacterial Strains** | | |
| *E. coli*: Strain OP50 | Caenorhabditis Genetics Center | N/A |
| *E. coli*: Strain HT115 (empty pL4440) | Miranda-Vizuete Lab | N/A |
| *E. coli*: Strain HT115 (RNAi clones) | Horizon Discovery (Cambridge, UK) | N/A |
| **Chemicals, Peptides and Recombinant Proteins** | | |
| Acetic acid | Sigma | Cat# A6283 |
| Acrylamide | Sigma | Cat# A3699 |
| Agar | Sigma | Cat# A1296 |
| APS | Sigma | Cat# A3678 |
| Arsenite | Sigma | Cat# S7400 |
| Beta-mercaptoethanol | Sigma | Cat# M7522 |
| Bleach | Household | NA |
| Boric acid | Sigma | Cat# B0252 |
| Bradford Reagent | Bio-Rad | Cat# 5000006 |
| Bromophenol blue | Sigma | Cat# 114391 |
| BSA | Sigma | Cat# A3059 |
| CaCl_2_ | Sigma | Cat# C1016 |
| Carbonyl Cyanide m-Chlorophenylhydrazone | Sigma | Cat# C2759 |
| Chloroform | Sigma | Cat# C0549 |
| Cholesterol | Sigma | Cat# C8667 |
| dATP | Thermo Fisher Scientific | Cat# R0141 |
| dCTP | Thermo Fisher Scientific | Cat# R0151 |
| dGTP | Thermo Fisher Scientific | Cat# R0161 |
| DTT | Sigma | Cat# Y00147 |
| dTTP | Thermo Fisher Scientific | Cat# R0171 |
| EDTA | Sigma | Cat# ED2SS |
| Ethanol | Sigma | Cat# E7023 |
| Fast SYBR™ Green Master Mix | Applied | 4385617 |
| Glycerol | Sigma | Cat# G6279 |
| Glycine | Sigma | Cat# G8898 |
| Isopropanol | Sigma | Cat# I9516 |
| K_2_HPO_4_ | Sigma | Cat# 3786 |
| KH_2_PO_4_ | Sigma | Cat# P9791 |
| Levamisole | Sigma | Cat# L9756 |
| Methanol | Sigma | Cat# 34860 |
| MgSO_4_ | Sigma | Cat# M7506 |
| Na_2_HPO_4_ | Sigma | Cat# S0876 |
| NaCl | Sigma | Cat# S9888 |
| NaClO | Sigma | Cat# 1056142500 |
| NaOH | Sigma | Cat# S5881 |
| NEM | Sigma | Cat# E3876 |
| Nitrocellulose Blotting membrane | GE Healthcare Life Sciences | Cat# 1060003 |
| Nystatin | Sigma | Cat# N3503 |
| Paraquat | Sigma | Cat# 856177 |
| Peptone | Sigma | Cat# 91249 |
| Phosphatase Inhibitor | Merck | P0044 |
| Ponceau S Staining Solution | Thermo Fisher Scientific | Cat# A40000279 |
| Protease Inhibitor Cocktail | Sigma | Cat# P8340 |
| Proteinase K | Sigma | Cat# 03115828001 |
| Random hexamer | Sigma | Cat# 100026484 |
| REDTaq® ReadyMix™ PCR Reaction Mix | Merck | R2523 |
| Ribolock RNase Inhibitor | Thermo Fisher Scientific | Cat# EO0381 |
| RNase free water | Sigma | Cat# W4502 |
| RT buffer | Sigma | Cat# Y02321 |
| SDS | Sigma | Cat# L3771 |
| Sodium oleate | Sigma | Cat# O7501 |
| Superscript II | Sigma | Cat# 100004925 |
| SYBR Green | Qiagen | Cat# 339347 |
| SYBR™ Safe DNA Gel Stain | Thermo Fisher | S33102 |
| TEMED | Sigma | Cat# T9281 |
| TMRM | Thermo Fisher Scientific | Cat# I34361 |
| Triton^TM^ X-100 | Sigma | Cat# T8787 |
| Trizma® base | Sigma | Cat# T1503 |
| TRIzol Reagent | Life Technologies | Cat# 15596018 |
| Tunicamycin | Sigma | T7765 |
| Tween 20 | Merck | P9416 |
| UltraPure ^TM^ Agarose | Sigma | Cat# 16500 |
| **Critical Commercial Assays** | | |
| MitoTracker^TM^ Red CMXRos | Thermo Fisher Scientific | Cat# M7512 |
| MitoSOX^TM^ Red mitochondrial superoxide indicator | Thermo Fisher Scientific | Cat# M36008 |
| Carboxy-H_2_DCFDA | Thermo Fisher Scientific | Cat# C2938 |
| Oil Red O | Sigma | O0625 |
| **Experimental Models: Organisms/Strains** | | |
| *C. elegans*: wild type | Caenorhabditis Genetics Center |  |
| *C. elegans: prdx-2(gk169) II* | Elizabeth A Veal | VE1, Backcrossed with *gk169* |
| *C. elegans: prdx-3(gk529) III* | Caenorhabditis Genetics Center | VC1151 |
| *C. elegans: prdx-6*(*tm4225*) *IV* | NBRP *C. elegans* (Japan) | TM4225, Backcrossed 6X with wild type |
| *C. elegans: drp-1(tm1108) IV* | Miranda-Vizuete Lab | CU6372 |
| *C. elegans: fzo-1(tm1133) II* | Miranda-Vizuete Lab | CU5991 |
| *C. elegans: prdx-2(gk169) II; prdx-6(tm4225) IV* | Miranda-Vizuete Lab | VZ1297 |
| *C. elegans: eat-3(tm1107) II* | Van-Raamsdonk Lab | JVR063 |
| *C. elegans: zcIs4[hsp-4p::GFP] V* | Miranda-Vizuete Lab | SJ4005 |
| *C. elegans: zcIs13[hsp-6p::GFP + lin-15 (+)] V* | Miranda-Vizuete Lab | SJ4100 |
| C. elegans: *zIs356[daf-16p::daf-16a/b::GFP + rol-6(su1006)]* | Miranda-Vizuete Lab | TJ356 |
| *C. elegans: dvIs19[(pAF15)gst-4p::GFP::NLS] III* | Caenorhabditis Genetics Center | CL2166 |
| *C. elegans: zcIs14[myo-3p::GFP(mit)]* | Caenorhabditis Genetics Center | SJ4103 |
| *C. elegans*: *unc-119(ed3); Ex[myo-3p::tomm20::Rosella; unc-119(+)]* | Tavernarakis Lab | IR2539 |
| *C. elegans: prdx-2(gk169) II; dvIs19[(pAF15)gst-4p::GFP::NLS] III* | This study | MCD22 |
| *C. elegans: prdx-6(tm4225) IV; dvIs19[(pAF15)gst-4p::GFP::NLS] III* | This study | MCD29 |
| *C. elegans: prdx-2(gk169) II; zcIs14[myo-3p::GFP(mit)]* | This study | MCD26 |
| *C. elegans: prdx-6(tm4225) IV; zcIs14[myo-3p::GFP(mit)]* | This study | MCD27 |
| *C. elegans: prdx-2(gk169) II; unc-119(ed3); Ex[myo-3p::tomm-20::Rosella; unc-119(+)]* | This study | MCD23 |
| *C. elegans: prdx-6(tm4225) IV; unc-119(ed3); Ex[myo-3p::tomm-20::Rosella; unc-119(+)]* | This study | MCD30 |
| *C. elegans: prdx-2(gk169) II; zIs356[daf-16p::daf-16a/b::GFP + rol-6(su1006)]* | This study | MCD25 |
| *C. elegans: prdx-6(tm4225) IV; zIs356[daf-16p::daf-16a/b::GFP + rol-6(su1006)].* | This study | MCD31 |
| **Oligonucleotides** | | |
| See Table S1 | | |
| **Software and Algorithms** | | |
| Prism 9.5.1 and prism 11 | GraphPad Software | RRID:SCR_002798 |
| ImageJ | NIH | RRID:SCR_003070 |
| Image Studio Lite | Image Studio Lite | RRID:SCR_013715 |
| CeleST | Christophe Restif et al., 2014 [2] | N/A |
| **Other** | | |
| EVOS M7000 Imaging System | Thermo Fisher | AMF7000 |
| Nanodrop 2000 | Thermo Fisher | N/A |
| Odyssey® Fc Imaging System | LI-COR Biosciences | OFC-1025 |

1. Olahova, M., et al., *A redox-sensitive peroxiredoxin that is important for longevity has tissue- and stress-specific roles in stress resistance.* Proc Natl Acad Sci U S A, 2008. **105**(50): p. 19839-44.

2. Restif, C., et al., *CeleST: computer vision software for quantitative analysis of C. elegans swim behavior reveals novel features of locomotion.* PLoS Comput Biol, 2014. **10**(7): p. e1003702.
